# A comparative UHPLC-QTOF-MS/MS-based metabolomics approach reveals the metabolite profiling of wolfberry sourced from different geographical origins

**DOI:** 10.1016/j.fochx.2024.101221

**Published:** 2024-02-10

**Authors:** Yanting Li, Xiaoying Wang, Yuping Sa, Liuyan Li, Weibiao Wang, Lingling Yang, Shuqin Ding, Gidion Wilson, Youyue Yang, Yue Zhang, Xueqin Ma

**Affiliations:** Department of Pharmaceutical Analysis, School of Pharmacy, Ningxia Medical University, 1160 Shenli Street, Yinchuan 750004, China

**Keywords:** *Lycium barbarum* L, Geographical origins, Metabolomics, Metabolites, UHPLC-QTOF-MS/MS

## Abstract

Wolfberry, known as Goji berry, is the fruit of *Lycium barbarum* L. (LB). As a famous functional food and TCM, the cost and efficacy of LB are closely linked to its geographical origin. The present study aimed to establish an effective method for distinguishing LB from different geographical origins. By employing UHPLC-QTOF-MS/MS combined with multivariate analysis, the metabolite profiling of LB (199 batches) obtained from Ningxia, Gansu, Qinghai, and Xinjiang, was evaluated. The results demonstrated that the method effectively distinguished LB from the four regions, with a total of 148 different metabolites being detected. Subsequent assessment using heat maps, Venn analysis, receiver operating characteristics curves and dot plots revealed 21 of these metabolites exhibited exceptional sensitivity and specificity, with under-curve values approaching 1, thus indicating their potential as biomarkers for LB. These findings strongly support the suitability of UHPLC-QTOF-MS/MS-based metabolomics as an effective approach to identify the source of LB.

## Highlights

• An LC-MS-based metabolomics approach reveals the metabolite profiling of *Lycium barbarum* L (LB).
• 148 differential metabolites were identified among LB samples sourced from different origins.
• 21 metabolites exhibited high sensitivity and specificity thus as potential biomarkers of LB.
• The longitude location plays a crucial role in explaining the variations among the four LB origins.
• The greatest disparity in metabolites was observed between NX and XJ.

## 1 Introduction

The friuit of *Lycium barbarum* L. (LB), commonly called wolfberry or goji berry, has been utilized for thousands of years as a health food and also a traditional Chinese medicine (TCM) (Lu et al., 2019, Szliszka et al., 2009). Two thousands years ago, LB was found beneficial for human health and recorded in the book “Divine Farmer’s Classic of Materia Medica” (Zhou, et al., 2021). Now, it is an important TCM, herbal medicine, and dietary supplement officially recorded in the Chinese pharmacopoeia, American Herbal Pharmacopoeia, European Pharmacopoeia, etc. As a popular and famous tonic herb with sweet taste and warming properties, LB is also extensively used in functional food and cosmetic industries across China, Korea, Japan, and other Southeast Asian countries for centuries. Presently, LB fruits and their associated products have gained a global reputation for their remarkable efficacy, consequently dominating a significant portion of the global market in the trade of Chinese herbal medicines, functional foods, and cosmetics.

Traditionally, according to the theory of TCM, LB is used to nourish the liver and kidneys, as well as replenish essence, thereby enhancing visual acuity. Nowadays, increasing attentions have been paid for its diverse pharmacological functions including enhancing immunity, anti-fatigue, antitumor, anti-inflammatory, antioxidant activity, and so on (Ma et al., 2022, Vidovic et al., 2022). Because of high nutritive value and therapeutic effects of LB worldwide, the corresponding bioactive components, including polysaccharides, phenolics, carotenoids, alkaloids, vitamins, amino acids, and fatty acids have gradually been uncovered (Zeng, et al., 2023). Consequently, LB cultivation has expanded extensively to meet market demands, particularly in the northern provinces of China, such as Xinjiang, Qinghai, Gansu, and Ningxia (Yao, et al., 2018). Notably, Zhongning city in Ningxia province is widely acknowledged as an ideal natural habitat for the cultivation of LB due to its unique geographical characteristics and regional climate (Li, et al., 2017). It is well-accepted that no matter the primary metabolites or the secondary metabolites of an herbal medicine, both of which are closely associated with the growth and development of the herbs, and are directly affected by environmental factors (Duan et al., 2022, Mi et al., 2020). As a result, the chemical composition of LB varies significantly across different regions, leading to variable quality levels. Concurrently, with the rising consumer apprehension regarding the provenance and geo-authenticity of LB, there is a clear and urgent need to develop a precise and reliable method for discriminating source of LB (Lv, et al., 2020).

Through the utilization of several indices or active constituents of LB, certain analytical techniques have been developed to assess either the geographical origin or the quality of LB, including high-performance liquid chromatography (HPLC) (Patsilinakos, et al., 2018), liquid chromatography-mass spectrometry (LC-MS) (Chang, et al., 2021), raman spectroscopy (Campmajo, et al., 2021), gas chromatography-mass spectrometry (GC–MS) (Liu, et al., 2020), carbon isotope (Meng, et al., 2019), 2D-CNN (Hao, et al., 2022), electronic nose (Li, et al., 2017), etc. These methods provided a wide range of insights into the origin and quality of LB. However, with scant research conducted on the primary and secondary metabolites derived from diverse sources, the metabolic profiles of LB from disparate origins remain largely unexplored. Consequently, a more comprehensive analysis of LB's metabolites, utilizing a precise method, is warranted.

Therefore, the present study aims to develop a precise and feasible method for screening differential metabolites and predicting the geographical origins of LB based on untargeted metabolomic analysis by UHPLC-QTOF-MS/MS combined with chemometrics. Briefly, a total of 199 LB samples were collected from 4 different geographical origins and analyzed via UHPLC-QTF-MS/MS, the resulting data was further analyzed using multivariate statistical analysis to identify differential metabolites, and the related metabolic pathway was also analyzed utilizing KEGG. Finally, the dot plots and receiver operating characteristic (ROC) curves were established to find biomarkers of different origins of LB. The findings prompted that UHPLC-QTOF-MS/MS based untargeted metabolomics merged with a multifactorial chemometric approach, emerged as a potent tool capable of not only screening credible markers but also predicting the origin of LB.

## 2 Materials and methods

### 2.1 Reagents and chemicals

Mass spectra grade methanol, acetonitrile, and formic acid were obtained from Fisher Scientific, Nepean, Canada. Ammonium formate was mass spectra grade purchased from Sigma-Aldrich. Double-distilled water was freshly prepared in our laboratory. A total of 199 batches of pre-dried LB were collected from four different regions of China from 2018 to 2020, including Qinghai (QH: 96.719684^◦^, 37.356337^◦^), Gansu (GS: 104.682515^◦^, 36.577096^◦^), and Ningxia (NX:105.691537^◦^, 37.497421^◦^), Xinjiang (XJ: 82.891346^◦^, 44.599663^◦^). LB samples were identified by Prof. Ling Dong (Department of Pharmacognosy, Ningxia Medical University), with the corresponding voucher specimens (numbered NYYP2020901-199) were preserved in the herbarium of pharmaceutical analysis. The detailed information of LB samples was presented in Supplementary Tab. S1.

### 2.2 Sample preparation

LB samples were first dried under 60℃ conditions for 24 h, powdered using a pulverizer (Zhejiang Yili Industry and Trade Co., Ltd., Zhejiang, China), and sieved through an 80-mesh sieve after cooling to ambient temperature (25 ± 5℃). After mixing with a blender, approximately 75 mg of LB powder was precisely weighed and put into 2 ml EP tube. Then, 1.5 ml of 90 % methanol were added to each sample. The samples were vortexed for 1 min, extracted using an Elmasonic P 120H ultrasonic bath (Elma, Germany) for 1 h at ambient temperature (25 ± 5℃), and centrifuged at 12,500*g* for 20 min at 25℃. Finally, the supernatant was injected into the UHPLC-MS/MS system for analysis.

In addition to 199 samples, each sample was weighed to 1 g and mixed evenly to prepare a mixed quality control (QC) sample. The preparation method was consistent with the test sample. The QC samples served for quality assurance and quality control. Prior to analyzing the LB sample, the instrument was equilibrated by injecting the QC sample ten times. Subsequently, one QC sample was examined after every ten samples in the UHPLC-QTOF-MS/MS running sequence.

### 2.3 UHPLC-QTOF-MS/MS conditions

Qualitative analysis was performed using an Agilent 1290 LC system (Agilent Technologies, Santa Clara, CA, USA) connected to an Agilent 6545 Q-TOF mass spectrometer (Agilent Technologies, Santa Clara, CA, USA). Agilent Mass Hunter Qualitative Analysis software (B.07.00) was used for MS control, data acquisition, and data analysis.

The separation column was ACQUITY UPLC HSS T3 (1.8 µm, 2.1 mm × 100 mm), with mobile phase was composed of water containing 0.1 % formic acid (solvent A) and acetonitrile containing 0.1 % formic acid (solvent B). The gradient elution program was set as follows: 0–5 min, 98 % A; 5–27 min, 94 % A; 27–30 min, 90 % A; 30–32 min, 85 % A; 32–38 min, 75 % A; 38–41 min, 48 % A; 41–51 min, 36 % A; 51–85 min, 2 % A. The flow rate was 0.2 ml/min, with column temperature was 30℃, and the injection volume was 2 μl.

MS/MS analyses were performed in both positive and negative ESI modes with the mass range set to *m*/*z* 50–1500 in full scan resolution mode at a scan rate of 30 scans per second. The ESI parameters were set as follows: sheath gas temperature 350℃, drying gas flow 12 L/min, capillary voltage 3.5 kV, fragmentor voltage 130 V, skimmer 65 V, and data were recorded in centroid mode. The ion scan was performed with a collision energy value of 40 eV under the same operating conditions as the primary MS scan.

### 2.4 Data analysis

The raw LC-MS/MS data (. d) were converted to (. mzML) format using Proteo Wizard software, then submitted and handled in Progenesis QI software (Nonlinear Dynamics, Newcastle, UK). The main parameters of Progenesis QI were set as follows: (1) data format: centroided data; (2) resolution (full width at half maximum): 60000; (3) data intensity filtering threshold: 0.1; (4) noise cancellation level: absolute intensity < 100; (5) retention time range (retention time limits): 0.5–80 min; (6) isotope distribution (isotope distribution). Delete the ions with an isotope distribution of 100. Data processing was done using the same parameters for different origins of LB to ensure that the analysis was done at the same level. When aligned, after summation ion analysis, features exhibiting the same *t*_R_ and *m*/*z* values correspond to the same eigenvariables, and the peaks were combined to generate a matrix of retention times (*t*_R_), *m*/*z* values, and peak intensities, exported in a CSV file format as *m*/*z*-t.

SIMCA 14.0 software (Umetrics, Umeå, Sweden) was used to carry out multivariate statistical analysis. Both unsupervised principal component analysis (PCA) and orthogonal partial least-squares discriminant analysis (OPLS-DA) were performed on the data. The metabolite data were log2 transformed (log2) to improve normality and mean centering before OPLS-DA analysis. A permutation test (200 permutations) was performed to avoid overfitting. A Venn analysis was then produced using an online tool (https://hiplot.com.cn/home/index.html) to discern similar metabolic profiles among LB samples from different origins. Besides, using the SPSS26 (IBM, Ehningen, Germany), ROC of each differential metabolite was established, and the area under curve of the ROC curve was calculated to evaluate the predictive ability of the discriminant markers (Rocchetti, et al., 2020). Meanwhile, heat maps were constructed to demonstrate the relative abundance of the significantly differential metabolites of LB from different regions (https://www.bioinformatics.com.cn). Dot plots were generated employing GraphPad Prism 9.4 to illustrate the distribution of the differential metabolites (GraphPad Software). Finally, the possible metabolic pathways were identified by searching the online Kyoto Encyclopedia of Genes and Genomes (KEGG) database (https://www.genome.jp/kegg/pathway.html).

### 2.5 Metabolite identification

The molecular features that exhibited the significant intergroup differences in the OPLS-DA models were selected for the identification. Ions with a variable importance in the projection (VIP) > 1.5, *p* < 0.05, and AUC > 0.7 were considered as candidate ions. The detected mass spectral features were searched using MetaScope search engine in Progenesis QI, utilizing various databases including our own LB SDF database and the Human Metabolome Database (https://hmdb.ca). A mass error of less than 50 ppm for primary MS (MS1) and less than 20 ppm for secondary MS (MS2) was applied during the search process. The identification of differential metabolites involved the consideration of retention times, accurate mass, and spectral fragments. By employing progenesis SDF Studio software (Nonlinear Dynamics), LB SDF database was established, and information on 312 different compounds was collected from relevant studies and reports of LB, the structures of these compounds were organized using the SciFinder database and then saved in. mol format. Statistical analysis was conducted using SPSS 26.0 software, with the results presented as mean ± SD. Differences between more than two groups were compared by one-way ANOVA, and *p* < 0.05 was considered as statistically significant.

## 3 Results and discussion

UHPLC-QTOF-MS/MS, characterized by high selectivity, sensitivity and accuracy, is proved as an effective and powerful tool for metabolomics analysis (Pan, et al., 2022). In the present study, a widely untargeted metabolite analysis based on UHPLC-QTOF-MS/MS was performed to explore the comprehensive metabolic profiling of LB obtained from NX, GS, QH, and XJ. The metabolic characteristics of each sample were examined, and the representative total ion chromatogram of LB samples from various geographic origins were illustrated in Supplementary Fig. S1, S2. A greater abundance of ions was observed in the positive ion mode compared to the negative ion mode. Intuitively, the metabolic fingerprint profiles of LB samples from different origins were similar, which brought great challenges for their geographical discrimination. Therefore, more data mining techniques were needed to scrutinize the classification potential of acquired metabolic fingerprints of each LB sample. Meanwhile, Supplementary Fig. S3 demonstrated the superimposition of the TIC of all the QC samples, which implied the high stability, reproducibility, and consistency of UHPLC-QTOF-MS/MS analysis.

Besides a stable and repeatable UHPLC-QTOF-MS/MS analysis being essential, chemometric analysis of generated untargeted metabolomic fingerprints data was another critical step for LB geographical origin traceability. Briefly, the raw LB data were firstly converted to mzML format using ProteoWizard software; then were subjected to peak extraction and alignment processes applying Progenesis QI software, and a total of 8143 and 3075 peak features in positive and negative ion modes were extracted, respectively; after removing the 100 isotope mass ion features, remaining 7314 and 2528 peak features in positive and negative ion modes, respectively.

**Fig. 1:**
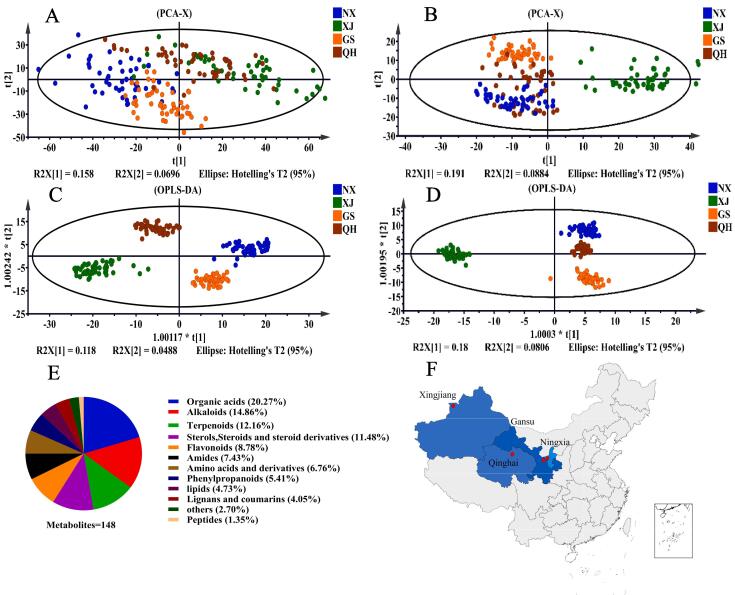
Multivariate statistical analysis and the classification of identified metabolites of LB sourced from 4 different origins. A and B: PCA score plots of LB in positive and negative ion modes, respectively; C and D: OPLS-DA score plots of LB in positive and negative ion modes, respectively. NX, GS, QH, XJ represent LB sample obtained from Ningxia, Gansu, Qinghai and Xinjiang, respectively. E is the classification of the 148 metabolites of LB samples. F indicates the primary distribution of the LB sample in China, as well as the sample under investigation in this study, and red dots denote the location of the LB source, which correspond to Zhongning city in Ningxia province, Baiyin city in Gansu province, Delingha city in Qinghai province, and Jinghe county in Xinjiang province. (For interpretation of the references to color in this figure legend, the reader is referred to the web version of this article.)

To efficiently identify the differential mass ion features of LB within the complex datasets mentioned above, PCA and OPLS-DA were performed to reduce the large amounts of data and to identify the most relevant differences among LB samples. Unsupervised PCA was firstly performed and the results were presented in Fig. 1 (A, B). In the positive ion mode, 4 groups of LB samples were not clear distinguished from each other; and in the negative ion mode, LB of NX, GS and QH were found to be clustered and partially overlapped, indicating a similarity between these samples from a negative metabolic perspective. This observation contrasted with the LB of XJ, suggesting a dissimilarity between these groups. Additionally, the R^2^X and Q^2^ of PCA were 0.652 and 0.455 in the positive ion mode, 0.673 and 0.488 in negative ion mode, respectively, indicating not satisfied repeatability and predictability of the model.

In contrast to the PCA model, OPLS-DA is a supervised model which can filter out the orthogonal variables in metabolites that are irrelevant to the categorical variables, thereby demonstrating superior classification capabilities compared to PCA (Xing, et al., 2021). The OPLS-DA score plots presented in Fig. 1 (C, D) exhibited distinct geographical clustering of LB, with no overlap observed among the different LB origins in both the positive and negative ion modes. The R^2^X, R^2^Y and Q^2^ values of OPLS-DA were 0.408, 0.936, 0.886 in the positive ion mode. Simultaneously, the values experienced a slight increase in the negative ion mode to 0.440, 0.938, and 0.909, respectively, indicating a high level of repeatability and predictability. Additionally, the findings from the 200-time permutation test provided evidence that there was no occurrence of over-fitting and the models employed were dependable (Supplementary Fig. S4, S5). All the aforementioned outcomes suggested that the untargeted metabolomic fingerprints data could be utilized to differentiate LB samples from different regions.

Based on the intuitive results of PCA and OPLS-DA, as depicted in Fig. 1A-D, LB samples of NX, GS and QH were clustered closer together than LB of XJ. Geographically, as illustrated in Fig. 1F, it was plausible that the observed phenomenon can be attributed to the location, particularly the longitude position. LB samples of NX were obtained from Zhongning city of Ningxia with the longitude coordinate was 37.497421^◦^, which was close to LB samples of GS (36.577096^◦^, Baiyin of Gansu) and QH (37.356337^◦^, Delingha of Qinghai) but far from samples of XJ (44.599663^◦^, Jinghe of Xinjiang) (Ren, et al., 2021). And although the latitudes of NX (105.691537^◦^) and GS (104.682515^◦^) were closer to each other compared to QH (96.719684^◦^), the LB samples from these three regions exhibited clustering, suggesting a weaker correlation between LB metabolites and latitude compared to longitude. These findings implied that geographical location, especially longitude, plays a crucial role in explaining the variations among the four LB origins.

**Fig. 2:**
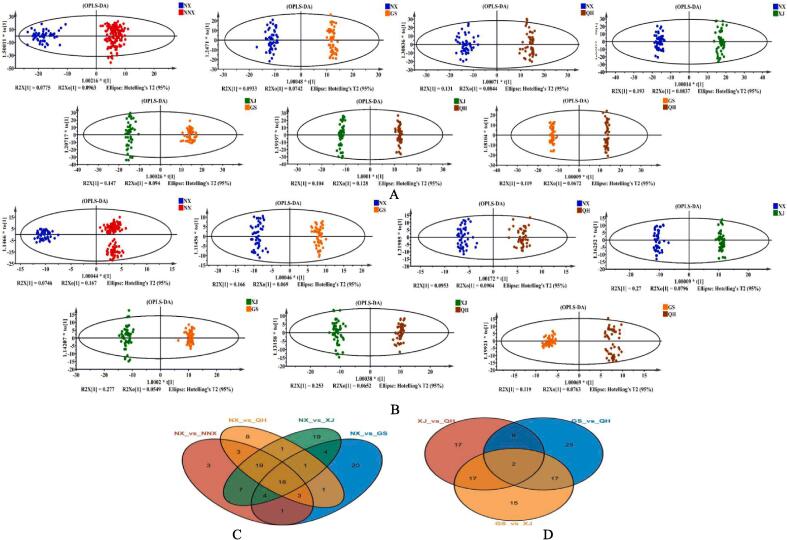
Pairwise OPLS-DA models and Venn diagram of shared and specific differential metabolites of LB samples from 4 origins. A and B: pairwise OPLS-DA models of LB samples in positive and negative ion modes, respectively; and the pairwise models include comparisons between NX vs NNX, NX vs GS, NX vs QH, NX vs XJ, XJ vs GS, XJ vs QH, and GS vs QH. C represents Venn diagram of shared and specific differential metabolites of LB originating from Ningxia compared with the other 3 regions, the comparison groups are NX vs NNX, NX vs GS, NX vs QH, and NX vs XJ. D illustrates Venn diagram of shared and specific differential metabolites of LB originating from the other 3 regions (excluding Ningxia), with the comparison groups being XJ vs GS, XJ vs QH, and GS vs QH.

To further identify and compare the specific differential metabolites of LB samples among these, a series of pairwise of OPLS-DA models were established, they were NX vs GS, NX vs QH, NX vs XJ, XJ vs GS, XJ vs QH, and GS vs QH. In addition, owing to the recognition of LB from Ningxia as an authentic and superior medicinal herb, the samples were divided into two new distinct groups: NX (consisting of LB samples from Ningxia) and NNX (consisting of LB samples from Gansu, Qinghai and Xinjing). The OPLS-DA results of the 7 pairwise comparisons mentioned above were presented in Fig. 2A, 2B. It was evident that each group was separated from the others in both positive and negative ion modes. The resulting values of R^2^Y and Q^2^ in this study were all greater than 0.88, indicating excellent fitness and predictability of the OPLS-DA model (Supplementary Tab. S2). Furthermore, the results from a 200-times permutation test demonstrated no evidence of overfitting, thereby confirming the reliability of the model (Supplementary Fig. S4, S5).

Based on the results of the OPLS-DA model, the identification of differential metabolites of LB was conducted using the following criteria: VIP > 1.5 for multivariate analysis, *p* < 0.05 for univariate statistical significance, and AUC > 0.7 for area under ROC curve. A combined analysis of positive and negative ion modes resulted in the structural characterization of a total of 148 differential metabolites (Supplementary Tab. S3, S4). Of these, 98 metabolites were identified in the positive ion mode, while 51 were detected in the negative ion mode. Only one metabolite was found to be present in both ion modes. Lycibarbarspermidine O was taken as an example to elucidate the mass spectrum in detail, as shown in Supplementary Fig. S6, and others were presented in Supplementary Fig. S7, S8. The identified metabolites were categorized into 12 categories, as illustrated in Fig. 1E, they were organic acids (20.27 %), alkaloids (14.86 %), terpenoids (12.16 %), sterols, steroids and steroid derivatives (11.48 %), flavonoids (8.78 %), amides (7.43 %), amino acids and derivatives (6.76 %), phenylpropanoids (5.41 %), lipids (4.73 %), lignans and coumarins (4.05 %), others (2.70 %), and peptides (1.35 %).

An integrated Venn diagram analysis was performed to ascertain unique and common metabolites among the 7 LB groups in both positive and negative ion modes combined. The results, depicted in Fig. 2C-D and Supplementary Tab. S3, S4, revealed a total of 73 distinct metabolites between NX and XJ, 58 between NX and NNX, 54 between NX and QH, 52 between NX and GS, 52 between GS and QH, 51 between XJ and GS, and 44 between XJ and QH were obtained. Notably, the greatest disparity in metabolites was observed between NX and XJ, while the smallest discrepancy was observed between XJ and QH, which was consistent with the findings of geographical location. The Venn diagram illustrated that there were 18 metabolites shared among the GX, QH, XJ and NX groups, while only 2 common metabolites were found among GS, QH and XJ groups. This observation highlighted significant disparities in the metabolite profiles across the four LB samples originating from different geographical locations. In addition, the results of heat maps of the differential metabolites among the four LB groups were displayed in Supplementary Fig. S9, S10, which these differential metabolites could be clearly distinguished based on their expression levels.

**Fig. 3:**
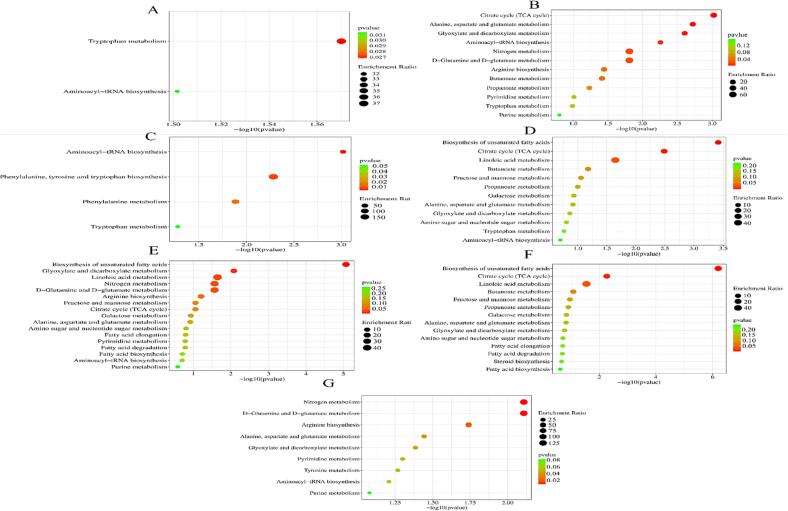
KEGG pathway analysis of differential metabolites between different origins of LB samples. A: NX vs NNX; B: NX vs GS; C: NX vs QH; D: NX vs XJ; E: XJ vs GS; F: XJ vs QH; G: GS vs QH. In the plot, each bubble (which represents a metabolic pathway) and abscissa indicate the size of the factors affecting the pathway (bigger bubbles represent bigger impacts). Bubble color indicates the *p*-value of the enrichment analysis, and lighter color illustrates lower enrichment.

After conducting KEGG enrichment analysis on the 148 differential metabolites, the associated metabolic pathways with *p* < 0.05 were identified. As shown in Fig. 3 and Supplementary Tab. S5, in the NX vs NNX comparison, there were noticeably enriched metabolic pathways associated with tryptophan metabolism, and aminoacyl-tRNA biosynthesis.

The comparison between NX and GS revealed significantly enriched metabolic pathways, including the citrate cycle (TCA cycle), alanine, aspartate and glutamate metabolism, glyoxylate and dicarboxylate metabolism. aminoacyl-tRNA biosynthesis, d-Glutamine and d-glutamate metabolism, nitrogen metabolism, arginine biosynthesis, butanoate metabolism. In the comparison of NX vs OH, the significant enrichment in metabolic pathways was observed in relation to aminoacyl-tRNA biosynthesis, phenylalanine, tyrosine and tryptophan biosynthesis, and phenylalanine metabolism. In the NX vs XJ comparison, there were noticeably enriched metabolic pathways associated with the biosynthesis of unsaturated fatty acids, citrate cycle (TCA cycle), and linoleic acid metabolism. When comparing XJ vs GS, notable enrichment was observed in metabolic pathways associated with the biosynthesis of unsaturated fatty acids, glyoxylate and dicarboxylate metabolism, linoleic acid metabolism, d-Glutamine and d-glutamate metabolism, and nitrogen metabolism. Similarly, in the comparison between XJ vs QH, enriched metabolic pathways were identified in relation to the biosynthesis of unsaturated fatty acids, citrate cycle (TCA cycle), and linoleic acid metabolism. Lastly, in the comparison between GS and QH, several metabolic pathways, including d-Glutamine and d-Glutamate metabolism, nitrogen metabolism, arginine biosynthesis, alanine, aspartate and glutamate metabolism, glyoxylate and dicarboxylate metabolism, exhibited significant enrichment. These findings suggested the existence of distinct metabolite profiles in the four LB different origins, potentially associated with their geographical origins.

Given LB from Ningxia is known as a geo-authentic medicinal material, the 58 differential metabolites between NX and NNX were selected for further analysis. These metabolites were categorized into 9 organic acids, 9 sterols, steroids and steroid derivatives, 9 alkaloids, 9 terpenoids, 6 amino acids and derivatives, 4 phenylpropanoids, 3 lignans and coumarins, 2 amides, 2 lipids, 2 flavonoids, 1 peptide and 2 other metabolites (Supplementary Tab. S3, S4). Notably, organic acid compounds in LB, including chlorogenic acid and ferulic acid, have been proved possessing antiviral, anti-tumor, anti-oxidation, anti-inflammatory and anti-thrombotic effects (Hu, et al., 2022). Alkaloids, such as *N*-methylcalystegine B2 in LB, was believed as potent competitive inhibitors of α-galactosidase and exhibited anti-diabetic and anti-tumor activities (Asano, et al., 1997). Citrostadienol is a compound of sterols in LB which had antioxidant property (Chen, et al., 2019), whereas lyciumionoside A is a terpenoid compound isolated from LB with anti-proliferative activity (Wang, et al., 2016). Recently, a series of free amino acids were used as metabolites markers to differentiate the LB of Ningxia (Zhongning city) from those originating from other provinces, suggested that amino acids played a crucial role for discriminating origins of LB (Gong, et al., 2022).

**Fig. 4:**
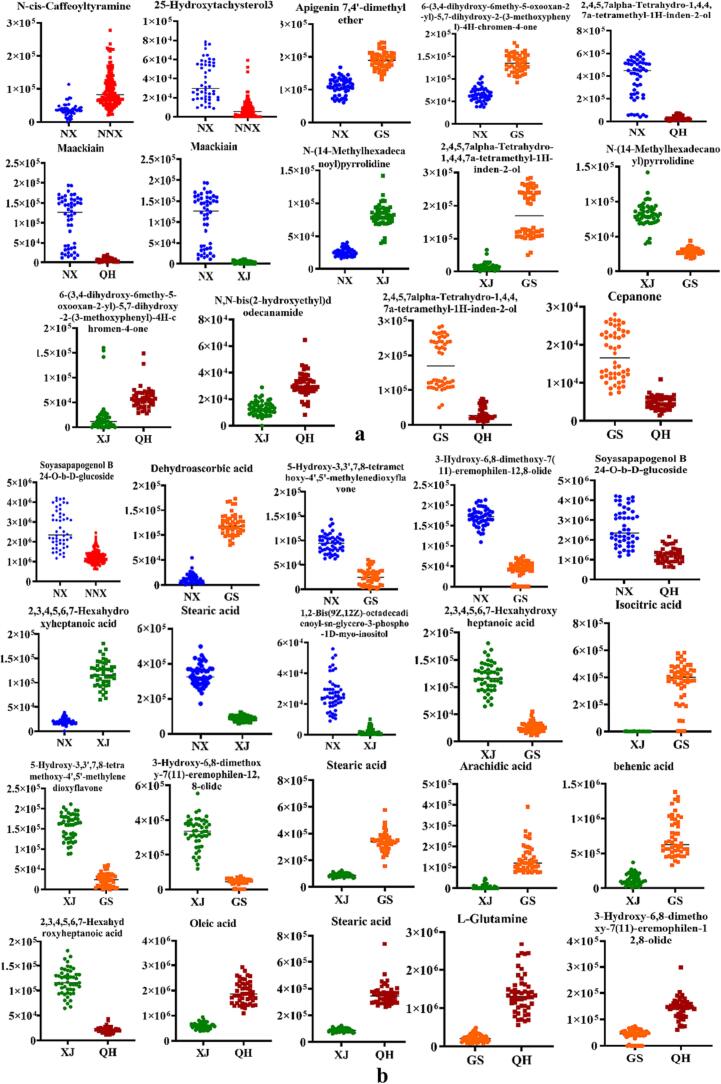
Dot maps of 21 metabolites from 7 pairwise comparisons in positive (a) and negative ion modes (b).

**Fig. 5:**
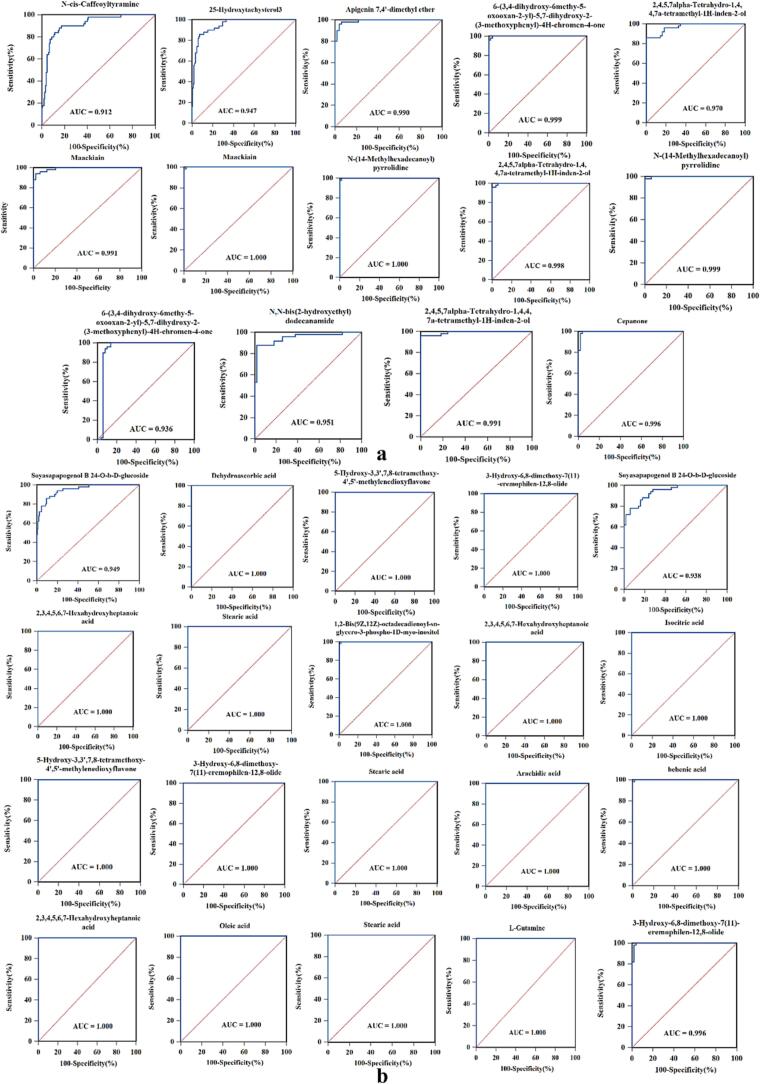
ROC curves of 21 metabolites from 7 pairwise comparisons in positive (a) and negative ion modes (b).

Although numerous differential metabolites were identified among various origins of LB, our specific focus was to determine potential markers for future LB discrimination. To achieve this, dot plots of all the different metabolites were established based on the relative quantification (Supplementary Fig. S11, 12, Tab. S3, S4). It was observed that the response levels of certain metabolites from various LB groups exhibited similarities or overlaps. The pronounced dispersion resulted in significant variations among the different LB groups, thereby influencing the potential application of biomarkers for distinguishing diverse LB samples. Therefore, firstly, we conducted an initial selection of metabolites that displayed comparatively higher differential response values between the 7 pairwise comparisons; and subsequently, metabolites with an area under curve value approaching 1 were identified as potential biomarkers of LB. Table 1 represented the screening results, indicating a total of 9 and 12 potential metabolites in the positive and negative ion modes, respectively. The dot diagrams and ROC curves were presented in Fig. 4, Fig. 5, respectively. Within Table 1, a total of 21 differential metabolites were found across the 7 comparison groups, with the specific counts being 3, 3, 4, 5, 5, 5 and 9 for the comparisons NX vs NNX, NX vs QH, GS vs QH, NX vs GS, NX vs XJ, XJ vs QH, and XJ vs GS, respectively. And these 21 differential metabolites were considered key metabolites for LB, comprising of 7 organic acids, 5 flavonoids, 2 amides, 2 others, 1 lipid, 1 sterols, steroids and steroid derivatives, 1 terpenoids, 1 amino acids and derivatives, as well as 1 alkaloid (Table 1). Organic acids and flavonoids were found to be the predominant metabolites. According to a study conducted by Y. Li, et al (Li, et al., 2022), there were notable disparities in the organic acids and amino acids of LB found in Qinghai and non-Qinghai provinces. These differences were likely attributed to various environmental factors such as ultraviolet radiation, soil composition, and altitude. In order to investigate the flavonoid metabolites and their associated gene expression levels in LB, R. Ma, et al (Ma, et al., 2023) employed transcriptome sequencing and metabolomics techniques, and the results revealed that quercetin compounds play a significant role in distinguishing LB from GS, NX, and QH. To sum up, our findings provided 21 potential markers for distinguishing LB from various sources, and these candidate biomarkers, which were screened to distinguish LB from different origins, also could be used as ideal chemical markers for the quality control purposes.

**Table 1:** 21 Potential biomarkers of LB with high sensitivity and specificity between different origins.

mode	compound	AUC						
		NNX vs NX	NX vs GS	NX vs QH	NX vs XJ	XJ vs GS	XJ vs QH	GS vs QH
Positive	*N*-*cis*-Caffeoyltyramine	0.912						
	25-Hydroxytachysterol3	0.947						
	Apigenin 7,4′-dimethyl ether		0.990					
	6-(3,4-dihydroxy-6methy-5-oxooxan-2-yl)-5,7-dihydroxy-2-(3-methoxyphenyl)-4H-chromen-4-one		0.999				0.936	
	Maackiain			0.991	1.000			
	2,4,5,7alpha-Tetrahydro-1,4,4,7a-tetramethyl-1H-inden-2-ol			0.970		0.998		0.991
	N-(14-Methylhexadecanoyl) pyrrolidine				1.000	0.999		
	N, *N*-bis (2-hydroxyethyl) dodecanamide						0.951	
	Cepanone							0.996
negative	Soyasapapogenol B 24-O-*b*-d-glucoside	0.949		0.938				
	5-Hydroxy-3,3′,7,8-tetramethoxy-4′,5′-methylenedioxyflavone		1.000			1.000		
	3-Hydroxy-6,8-dimethoxy-7(11)-eremophilen-12,8-olide		1.000			1.000		0.996
	Dehydroascorbic acid		1.000					
	1,2-Bis(9Z,12Z)-octadecadienoyl-*sn*-*glycero*-3-phospho-1D-myo-inositol				1.000			
	2,3,4,5,6,7-Hexahydroxyheptanoic acid				1.000	1.000	1.000	
	Isocitric acid					1.000		
	Stearic acid				1.000	1.000	1.000	
	Arachidie acid					1.000		
	Behenic acid					1.000		
	Oleic acid						1.000	
	l-Glutamine							1.000

## 4 Conclusion

LB is widely cultivated in China, and the prospect was remarkable, thus the findings of this study are particularly noteworthy. The metabolite profiling of LB from four main production regions (Ningxia, Gansu, Qinghai, and Xinjiang) were analyzed using UHPLC-QTF-MS/MS non-targeted metabolomics in the present study. A series of multivariate analysis revealed significant differences among LB samples based on the distinct geographical origins, and a total of 148 differential metabolites belonging to 12 categories have been identified. Notably, through the establishment of ROC and dot plots, we identified 21 metabolites exhibiting high sensitivity and specificity in tracing the origin of LB. Therefore, the findings offer a valuable reference for assessing source traceability of LB.

## CRediT authorship contribution statement

**Yanting Li:** Writing – original draft, Formal analysis, Data curation, Conceptualization. **Xiaoying Wang:** Writing – review & editing, Validation, Resources, Methodology, Data curation. **Yuping Sa:** Methodology, Investigation, Data curation. **Liuyan Li:** Writing – review & editing, Validation, Resources, Methodology, Data curation. **Weibiao Wang:** Supervision, Resources, Formal analysis. **Lingling Yang:** Visualization, Validation, Methodology, Investigation. **Shuqin Ding:** Supervision, Resources, Methodology. **Gidion Wilson:** Writing – review & editing, Data curation. **Youyue Yang:** Methodology, Investigation, Data curation. **Yue Zhang:** Methodology, Investigation, Data curation. **Xueqin Ma:** Writing – review & editing, Supervision, Project administration, Funding acquisition, Conceptualization.

## Declaration of competing interest

The authors declare that they have no known competing financial interests or personal relationships that could have appeared to influence the work reported in this paper.

## Data Availability

Data will be made available on request.
