# Supplementary material for: A comparative UHPLC-QTOF-MS/MS-based metabolomics approach reveals the metabolite profiling of wolfberry sourced from different geographical origins
Source: Food Chem X. 2024 Feb 10;21:101221. doi: 10.1016/j.fochx.2024.101221 (PMC10877177; doi:10.1016/j.fochx.2024.101221)
Supplement: Supplementary data 1 [file mmc1.docx]

**Legends of Supplementary Figures**

**Fig. S1.** Representative TIC of LB sample from different geographical origins in the positive ion mode. (A) NX, (B) GS, (C) QH, (D) XJ.

**Fig. S2.** Representative TIC of LB sample from different geographical origins in the negative ion mode. (A) NX, (B) GS, (C) QH, (D) XJ.

**Fig. S3.** The overlap of TIC of QC sample in the positive and negative ion modes. (A) positive ion mode, (B) negative ion mode.

**Fig. S4.** Permutation tests of LB sample of 4 origins in the positive ion mode. (A) LB of 4 origins, (B) NX vs NNX, (C) NX vs GS, (D) NX vs QH, (E) NX vs XJ, (F) XJ vs GS, (G) XJ vs QH, (H) GS vs QH.

**Fig. S5.** Permutation tests of LB sample of 4 origins in the negative ion mode. (A) LB of 4 origins, (B) NX vs NNX, (C) NX vs GS, (D) NX vs QH, (E) NX vs XJ, (F) XJ vs GS, (G) XJ vs QH, (H) GS vs QH.

**Fig. S6.** MS/MS spectra and principal cleavage pathways of Lycibarbarspermidine O.

**Fig. S7.** MS/MS spectra of the tentative identification metabolites in the positive ion mode by using Progenesis QI software.

**Fig. S8.** MS/MS spectra of the tentative identification metabolites in the negative ion mode by using Progenesis QI software.

**Fig. S9.** Heat maps of differential metabolites of the 7 comparison groups in the positive ion mode. (A) NX vs NNX, (B) NX vs GS, (C) NX vs QH, (D) NX vs XJ, (E) XJ vs GS, (F) XJ vs QH, (G) GS vs QH.

**Fig. S10**: Heat maps of differential metabolites of the 7 comparison groups in the negative ion mode. (A) NX vs NNX, (B) NX vs GS, (C) NX vs QH, (D) NX vs XJ, (E) XJ vs GS, (F) XJ vs QH, (G) GS vs QH.

**Fig. S11.** Dot maps of differential metabolites in the positive ion mode.

**Fig. S12.** Dot maps of differential metabolites in the negative ion mode.

**Fig. S1**

**
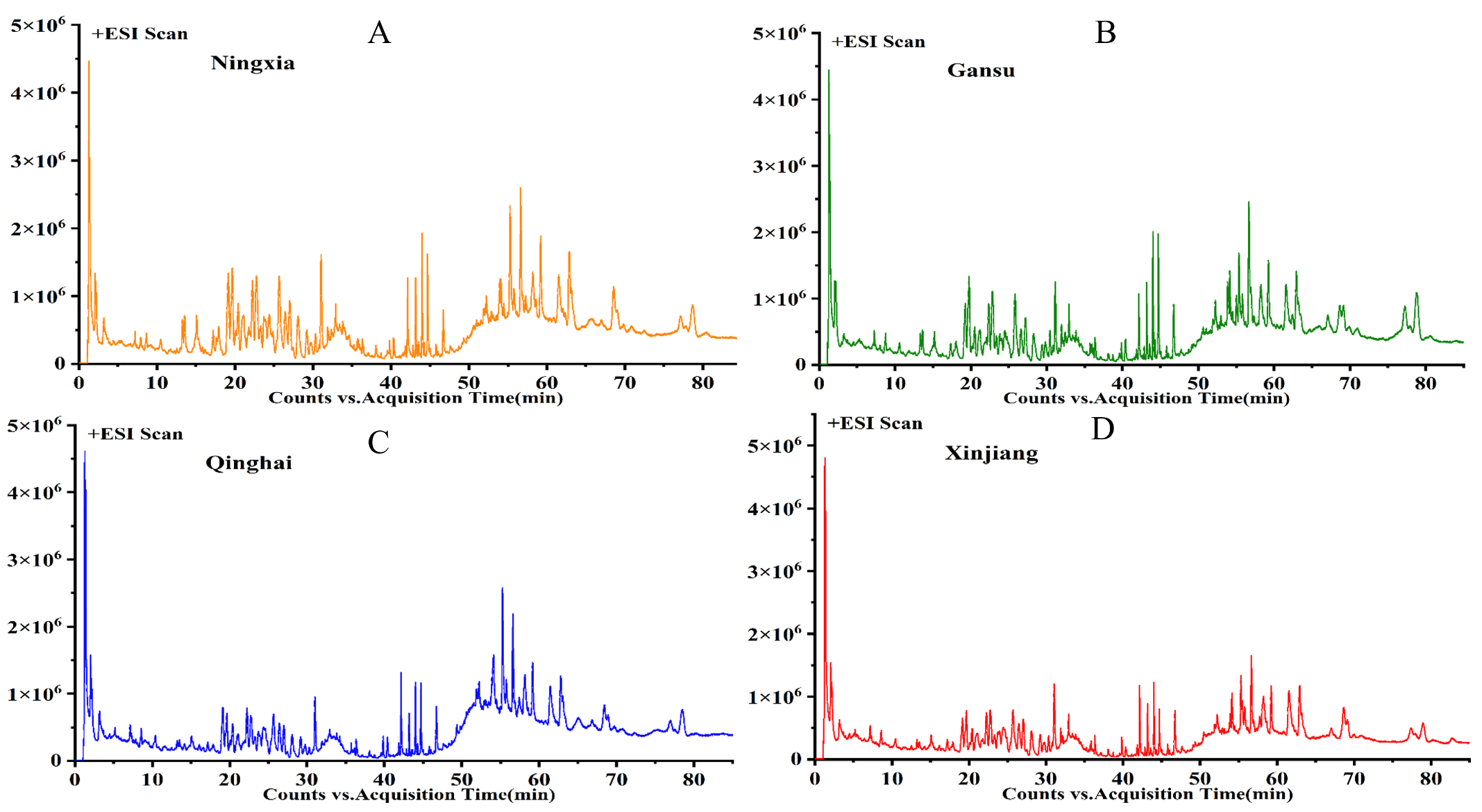
**

**Fig. S2**

**
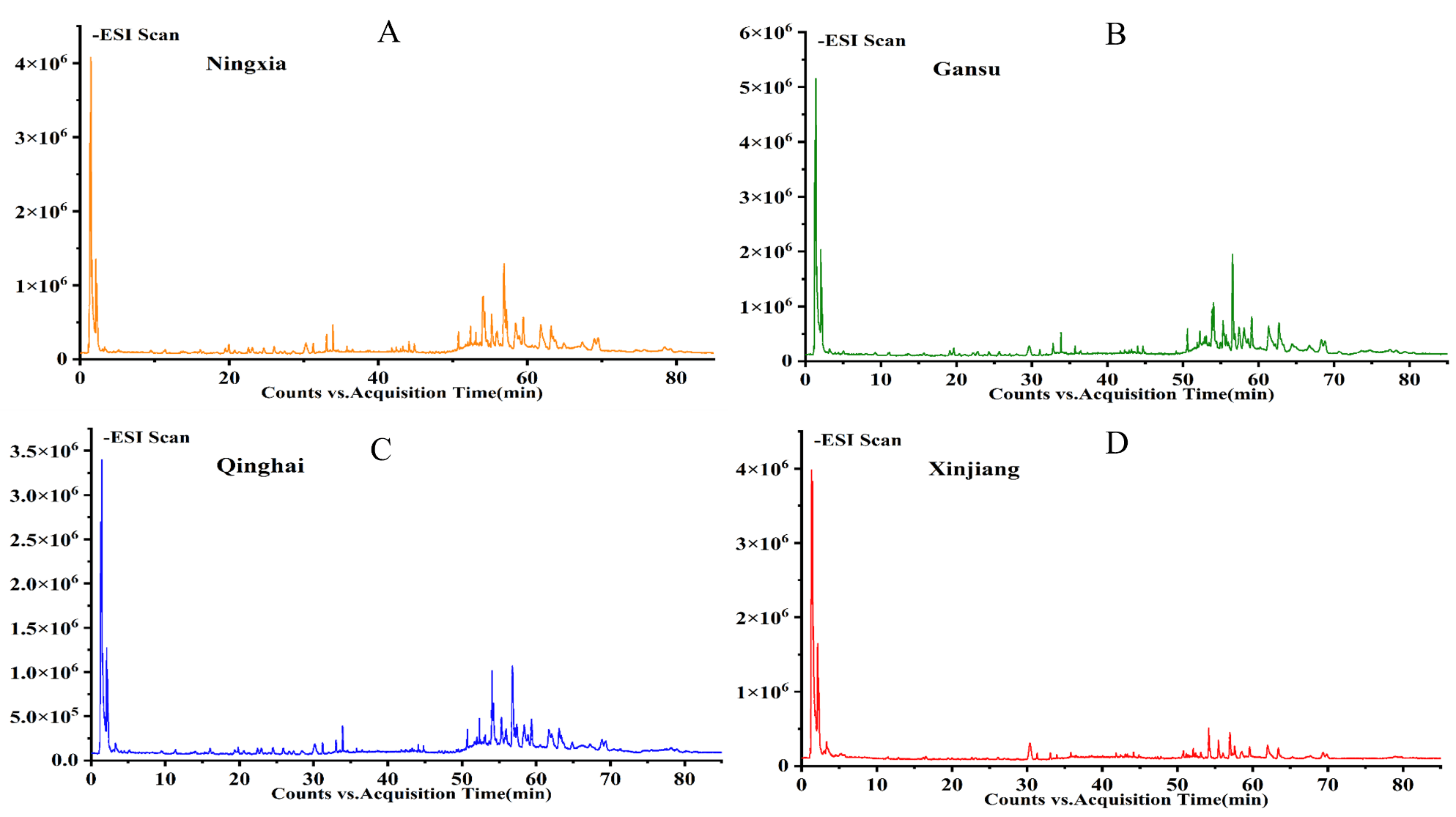
**

**Fig. S3**

**
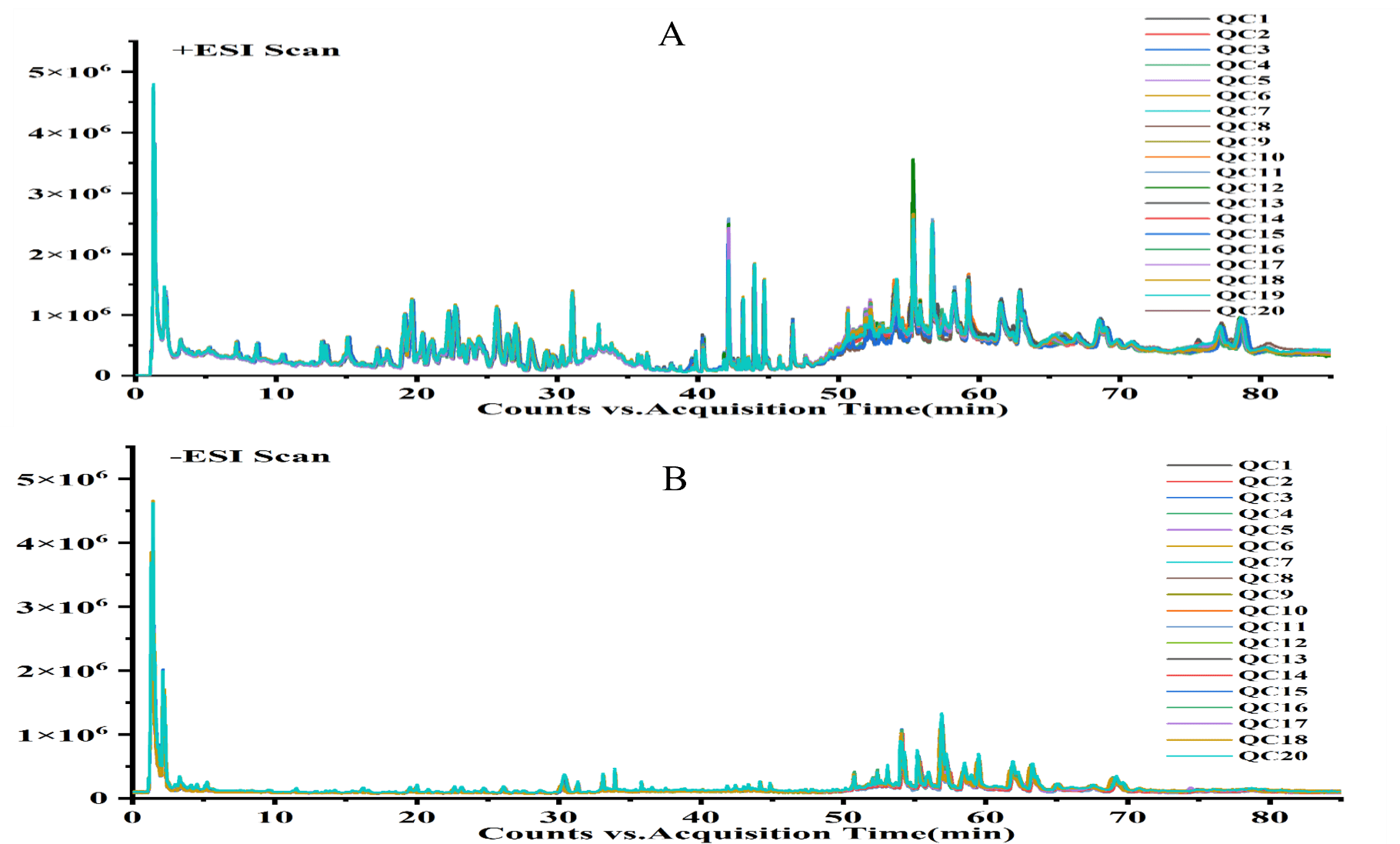
**

**Fig. S4**


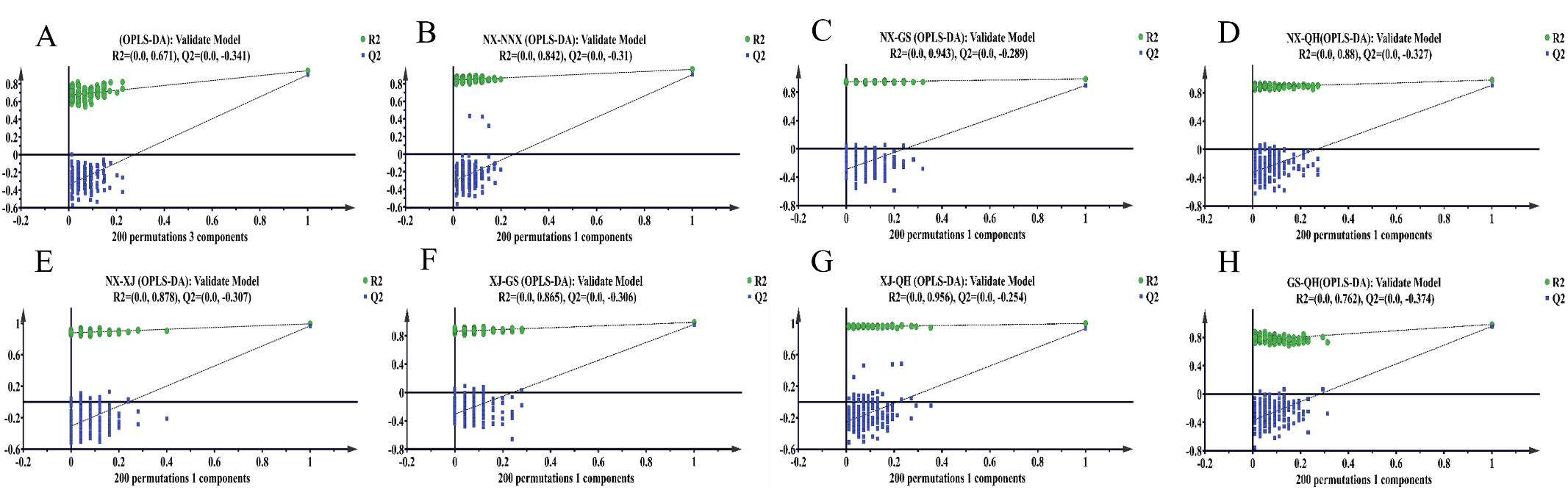


**Fig. S5**


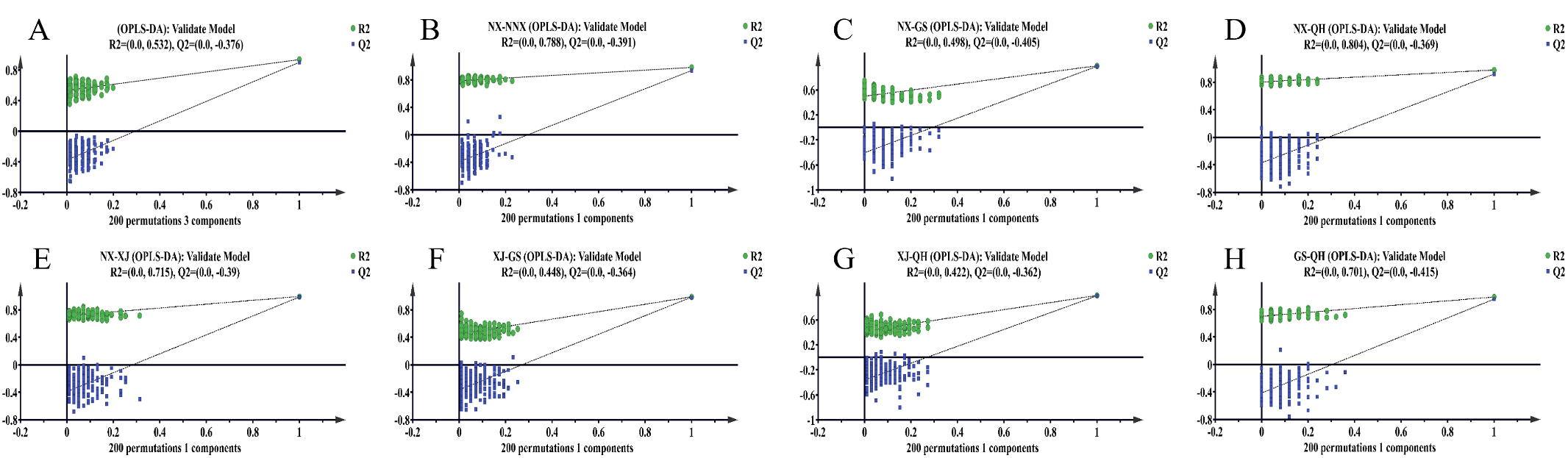


**Fig. S6**

**
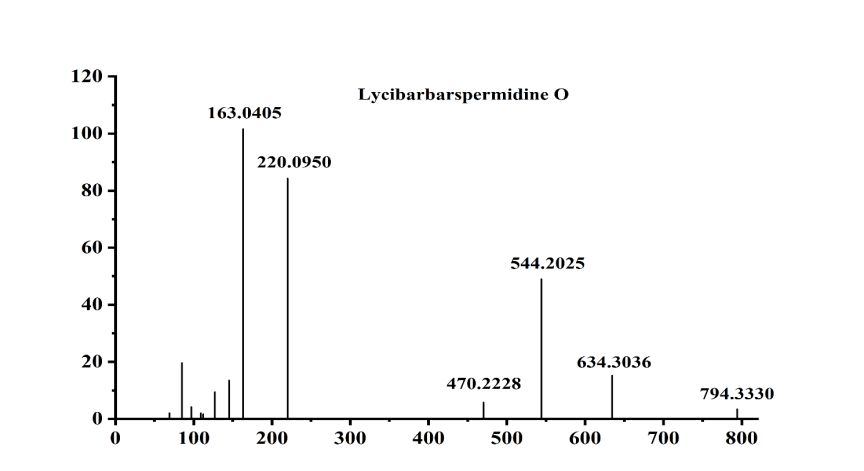
**

**
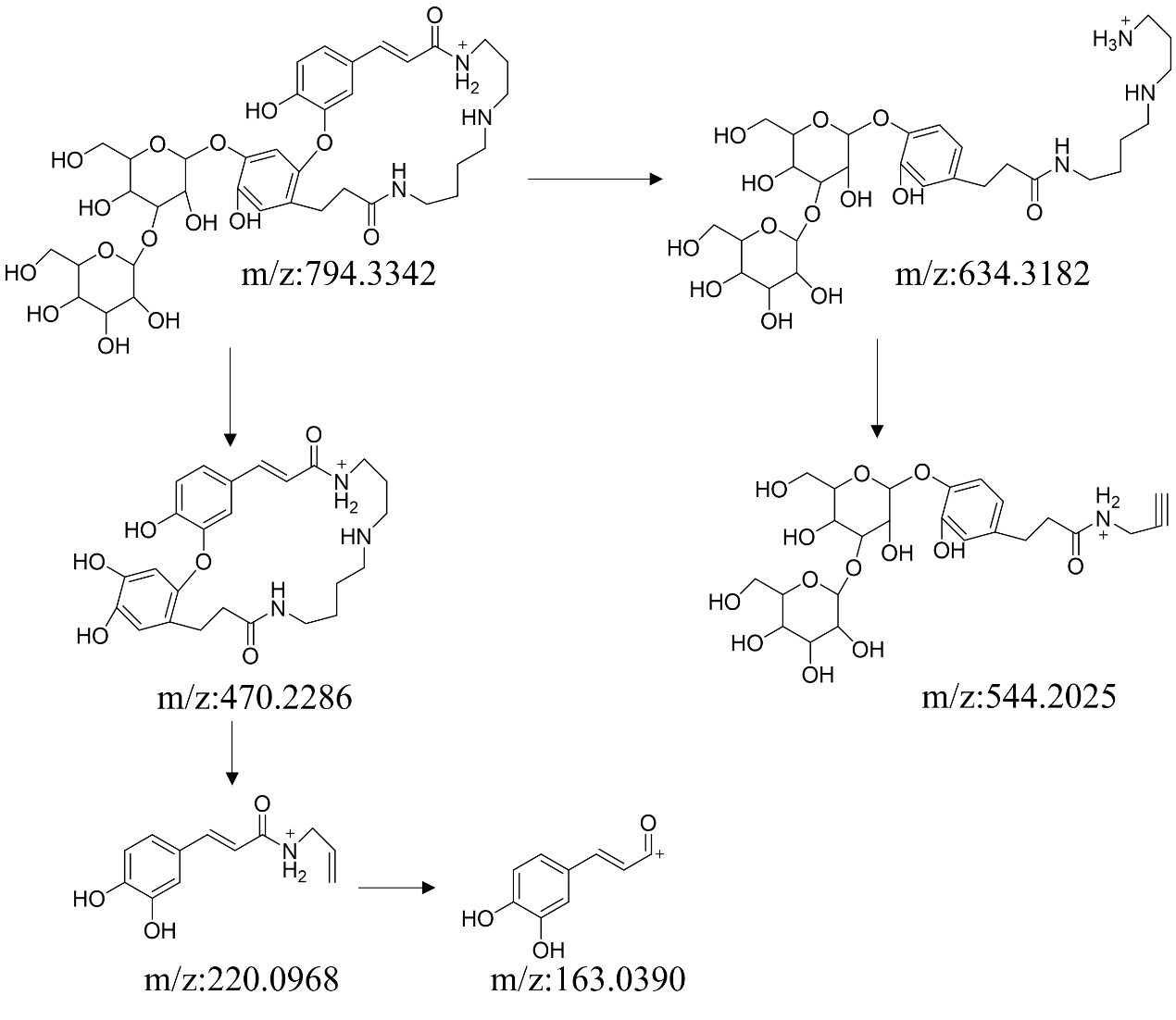
**

**Fig. S7**


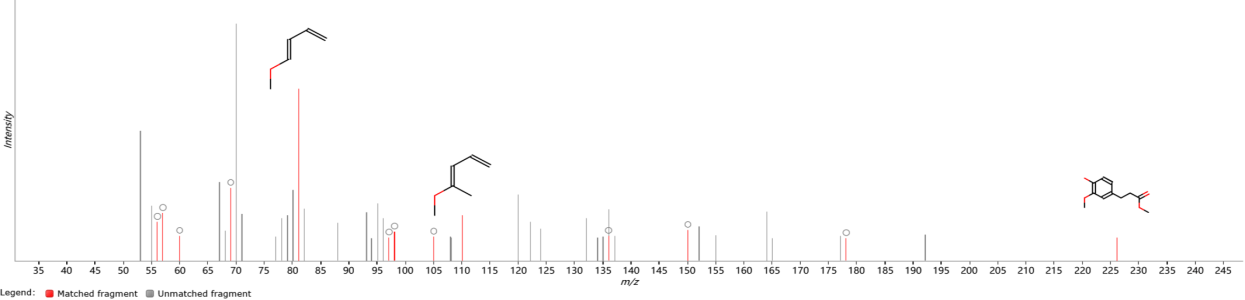


**Ethyl dihydroferulate**


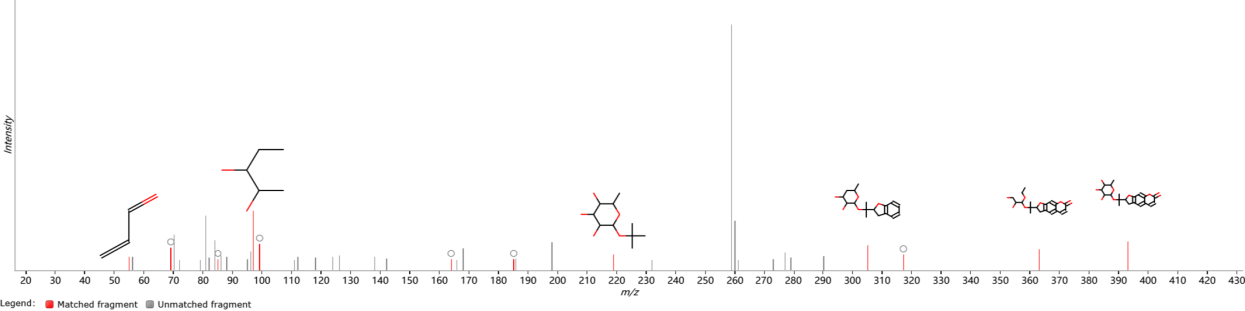
**Marmesin rhamnoside**


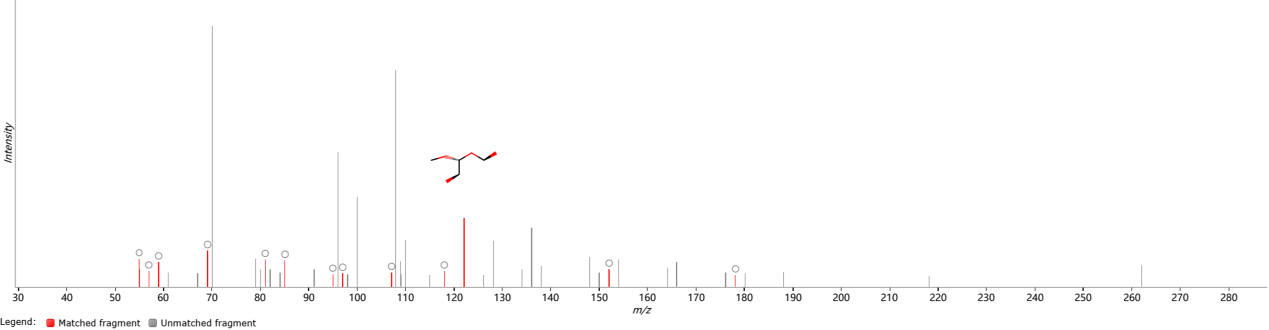


**1-O-methyl-4-O-p-E-coumaroyl-α-L-rhamnopyranoside**


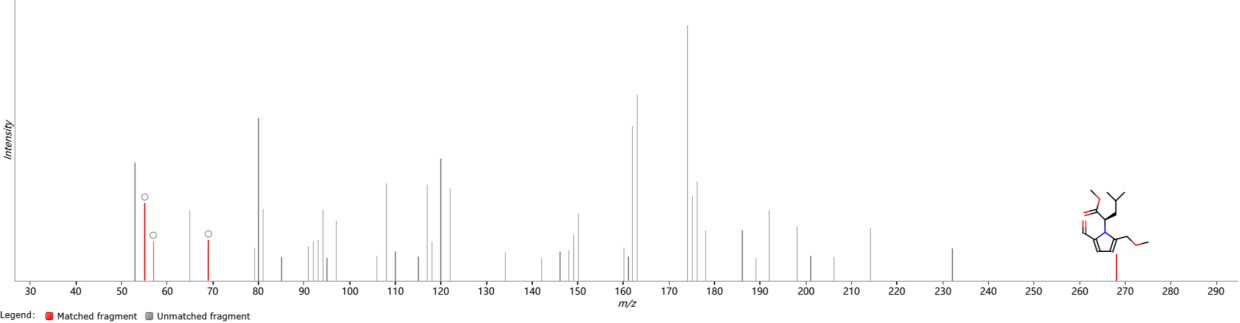


**Methyl (2R)-[2-formyl-5- (methoxymethyl)-1H-pyrrol1-yl]-4-methyl-pentanoate**


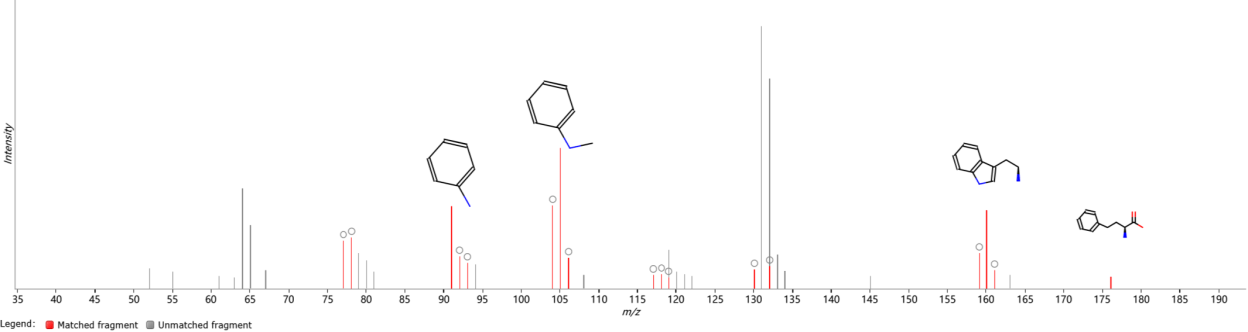


**Tryptophan**


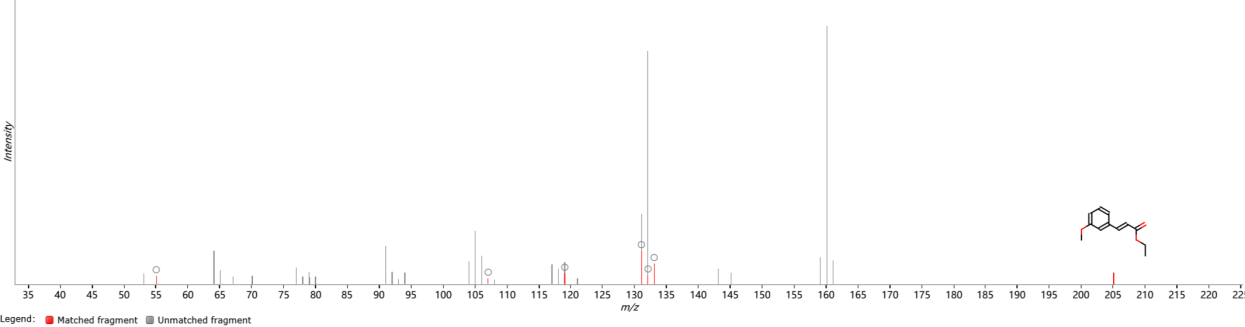
 **EthylE-ferulate**


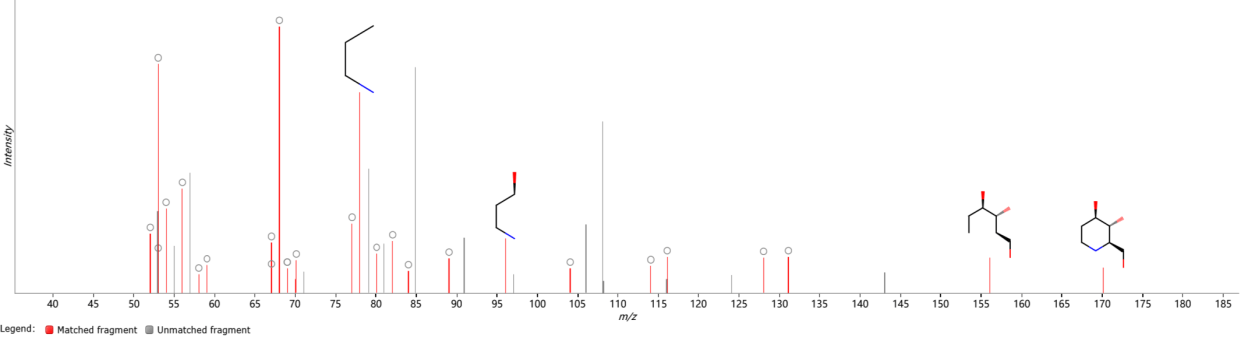


**Fagomine**


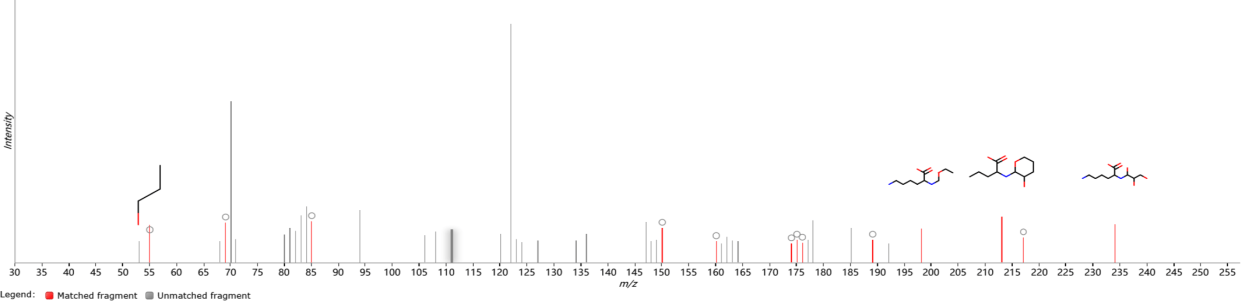


**N2-Galacturonyl-L-lysine**


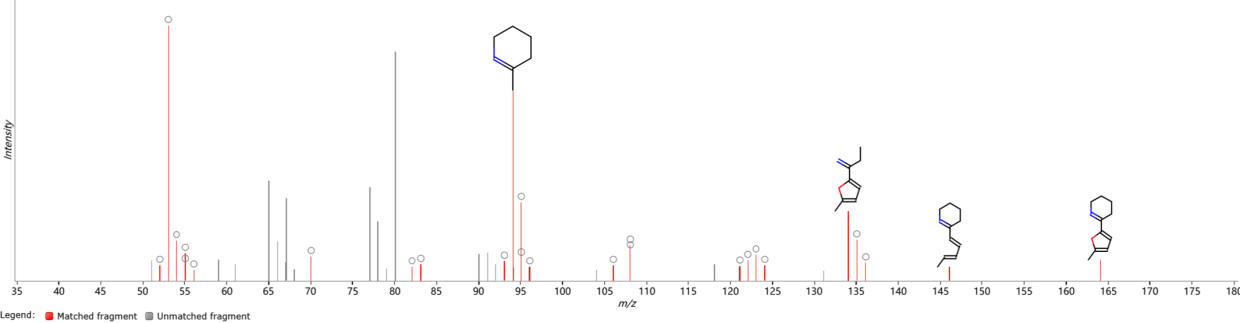
**2,3,4,5-Tetrahydro-6-(5-methyl-2-furanyl)pyridine**


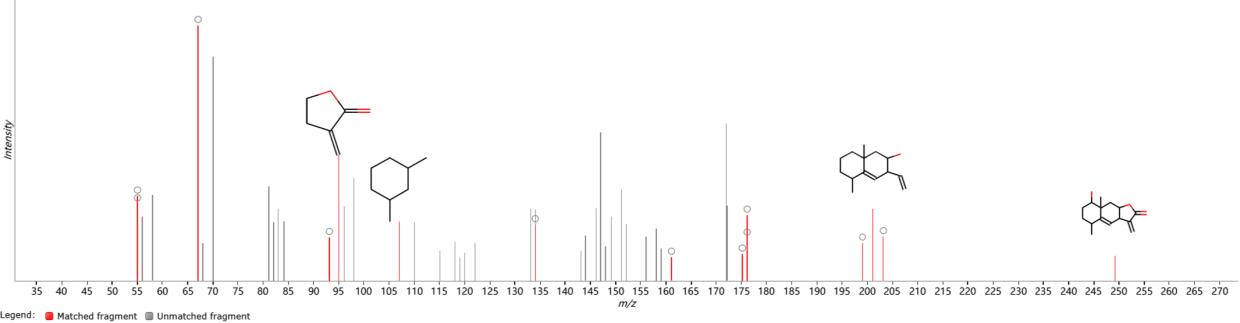
**1beta-Hydroxyalantolactone**


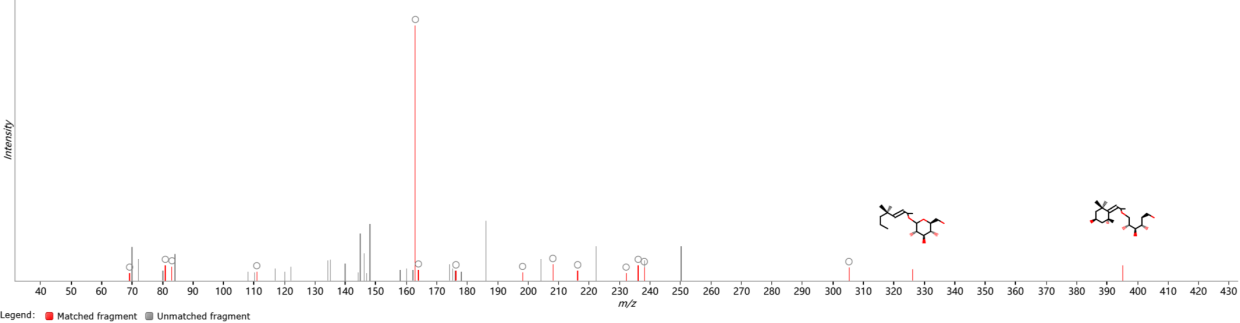


**3-(2,4-Dihydroxy-2,6,6-trimethylcyclohexylidene)-1-methylprop-2-enyl-β-D-glucopyranoside**


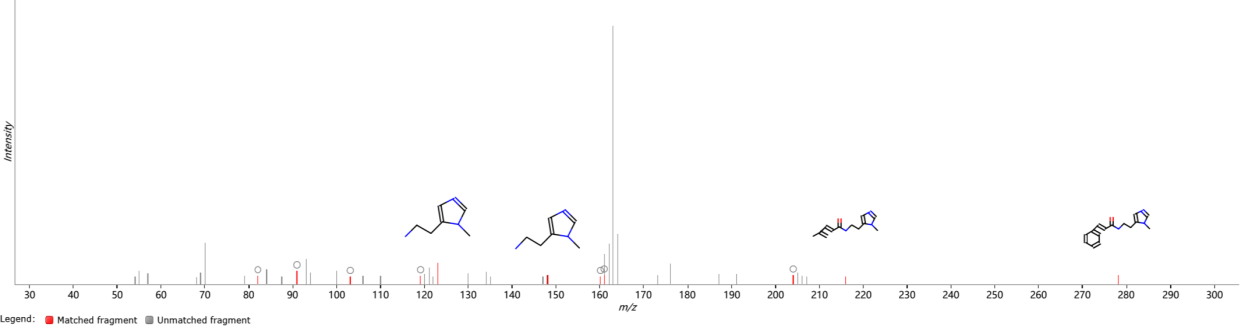
**Na-[(E)-Cinnamoyl]-N1-methylhistamine**


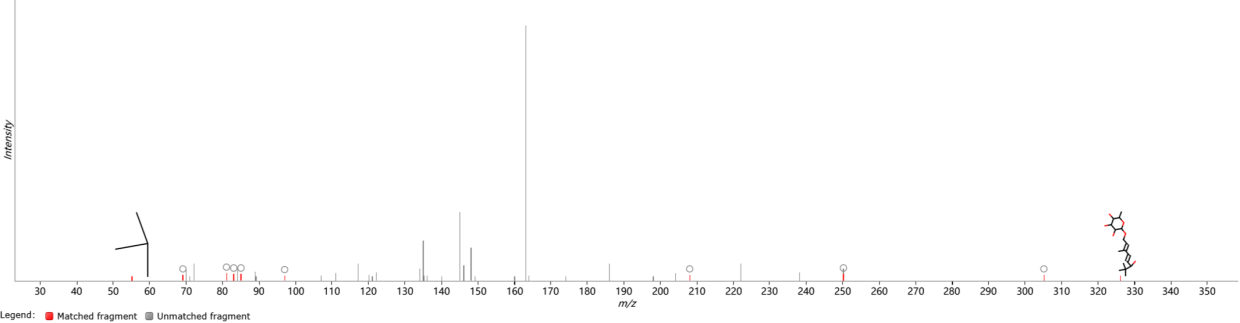
**Abscisic alcohol 11-glucoside**


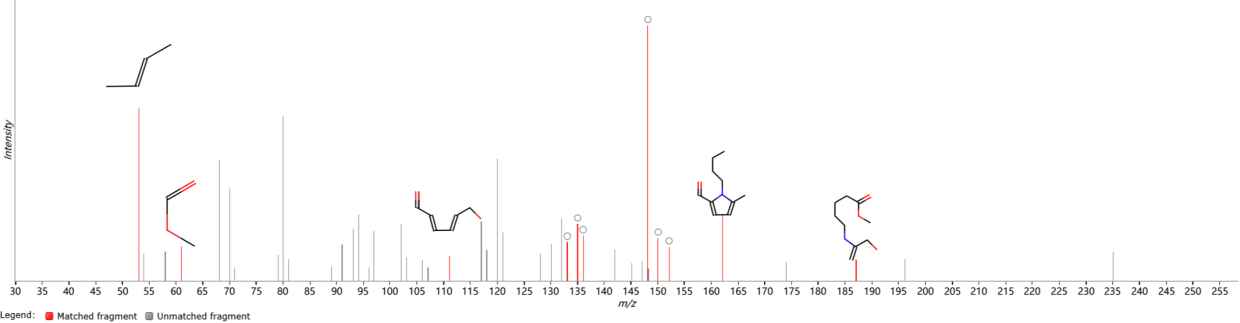
**Methyl 4-[2-formyl-5-(hydroxy-methyl)-1H-pyrrol-1-yl]-butanoate**


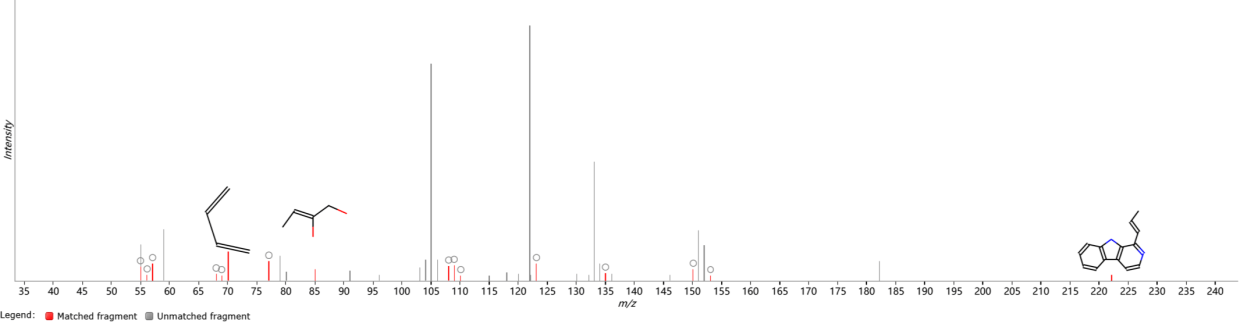
**Perlolyrine**


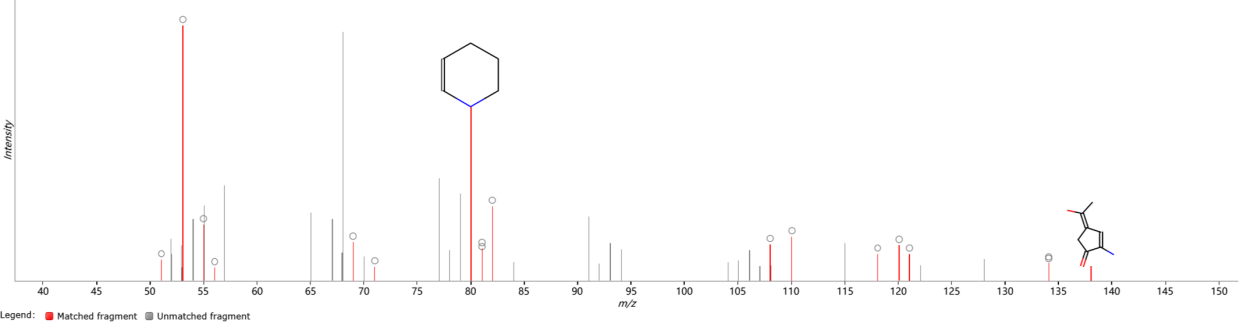


**1,2,3,4,5,6-Hexahydro-5-(1-hydroxyethylidene)-7H-cyclopenta[b]pyridin-7-one**


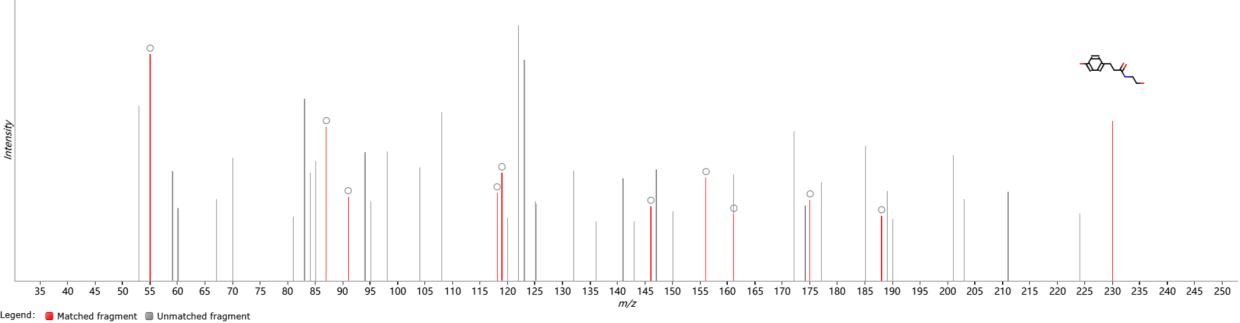


**Tyrosyl-Serine**


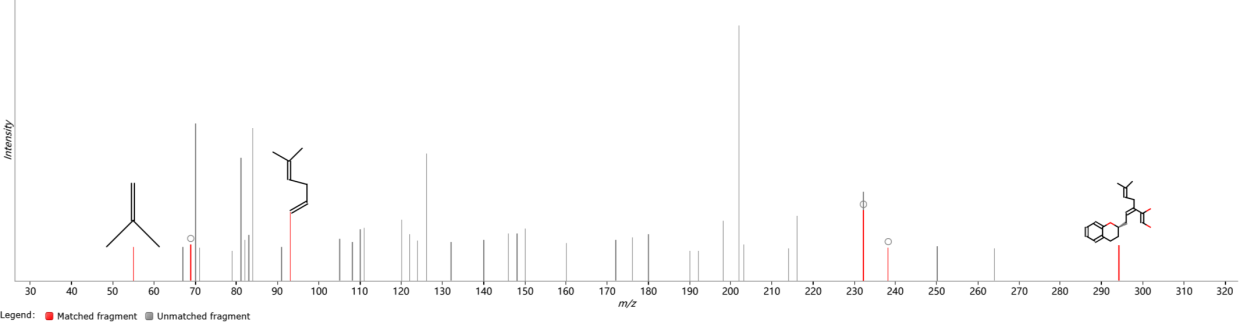
**Kcazinol A**


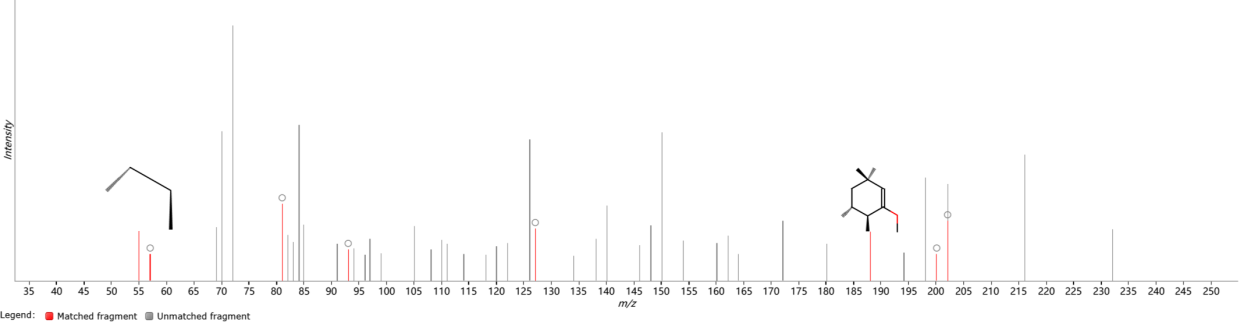
**Lyciumionoside A**


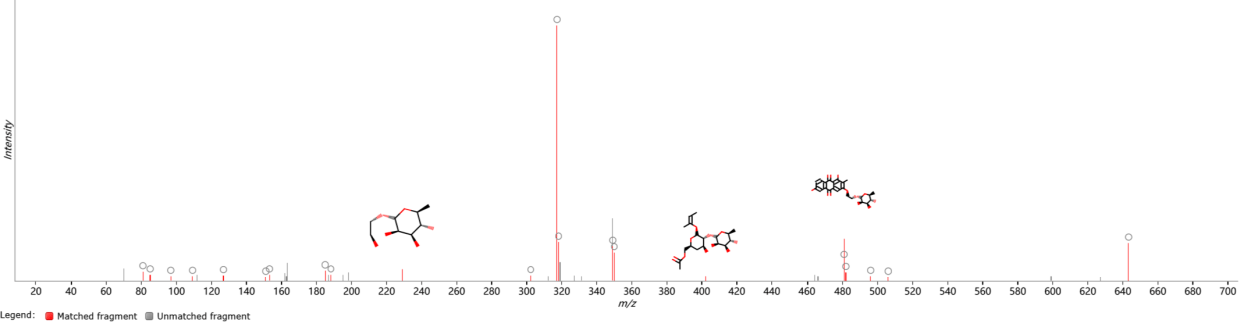
**3-O-(2-O-a-L-Rhamnopyranosyl-6-O-acetyl-β-D-glucopyranosyl)-6-hydroxyrubiadin**


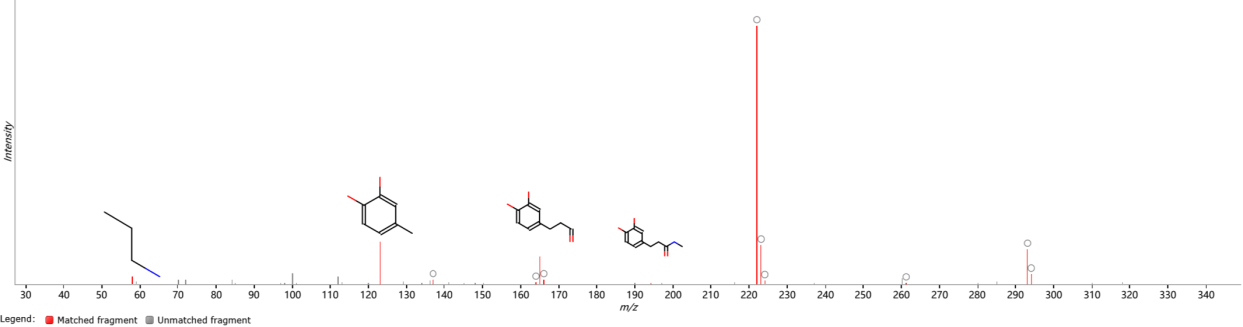
**Kukoamines B**


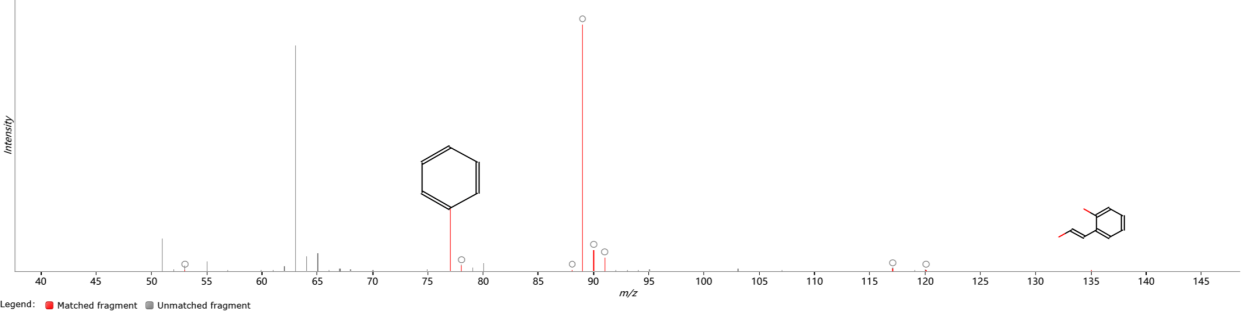
**3 Hydroxycoumarin**


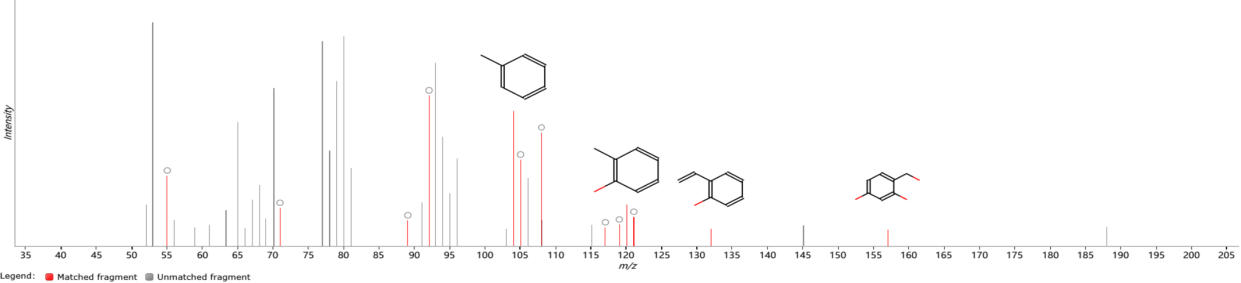
**7-hydroxy-6(hydroxymethyl)-2H-chromen-2-one**

| 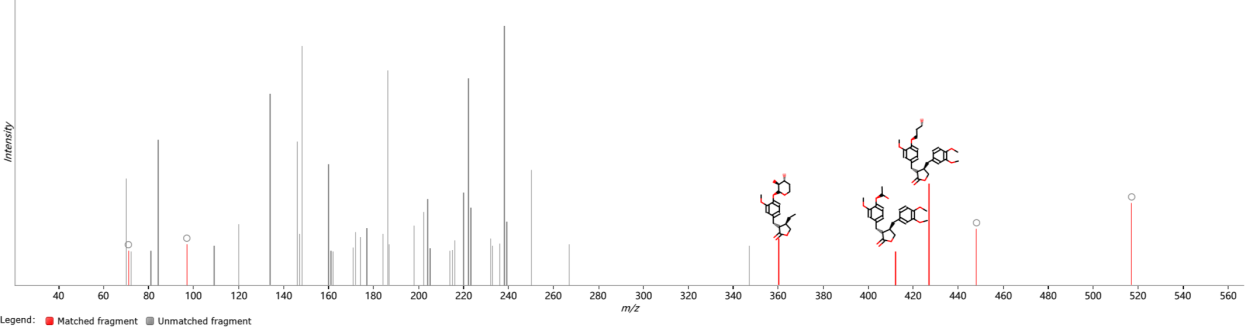 |
| --- |
| **Arctiin** 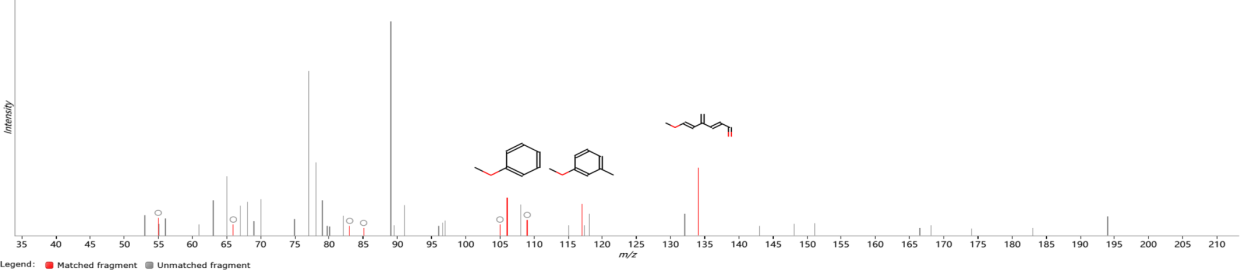**E-ferulic acid**  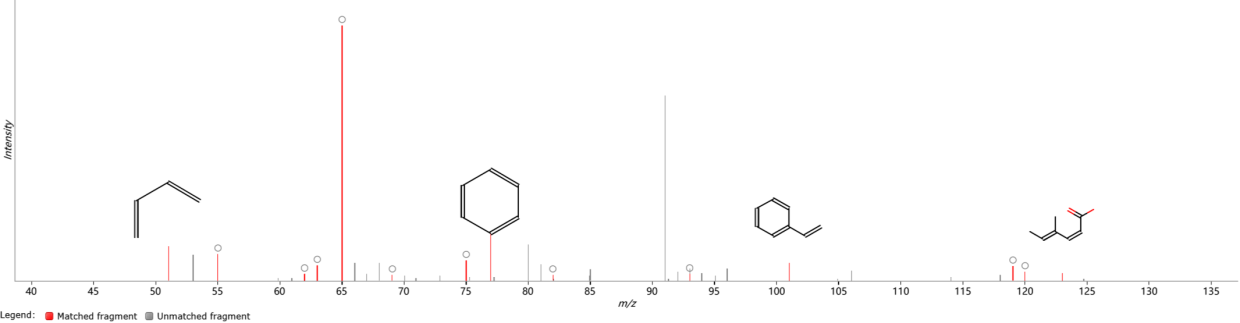 |

**Z-p-coumaric acid**
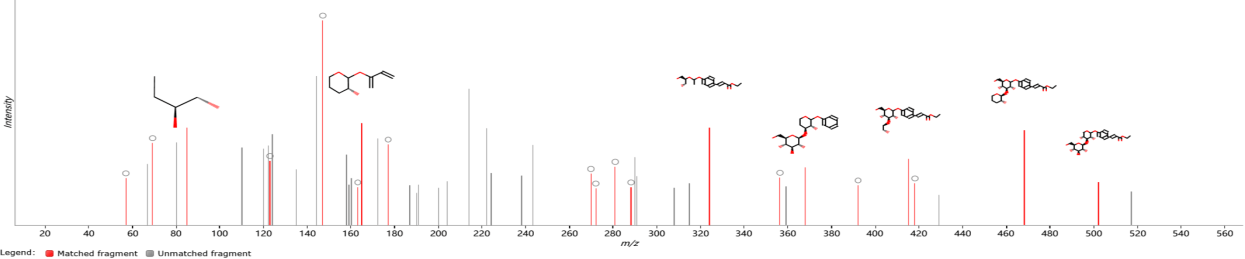
 **lycibarbarphenylpropanoid F**
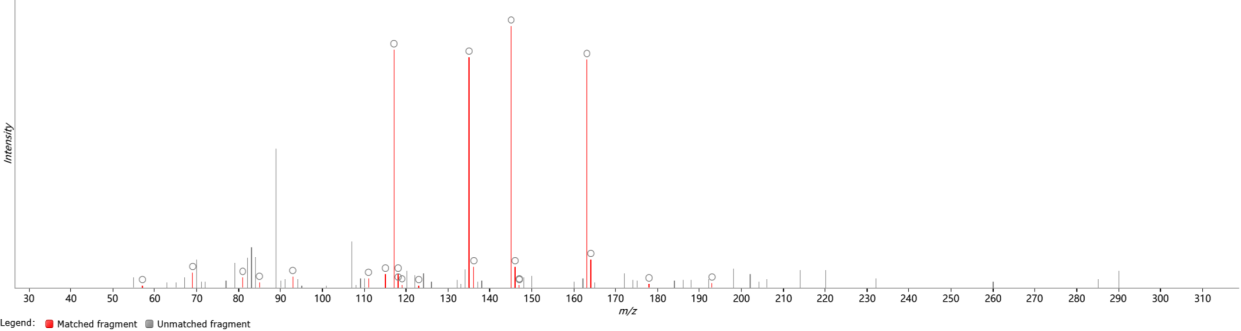
 **Chlorogenic acid**

| **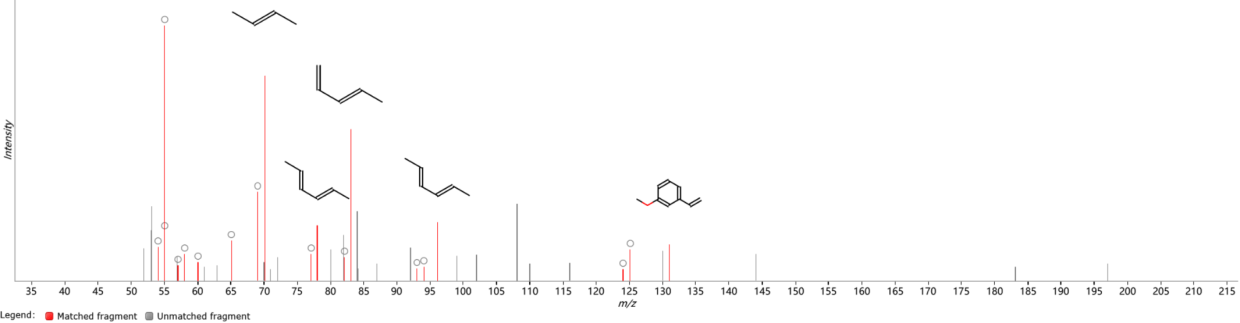coniferol** |
| --- |
| 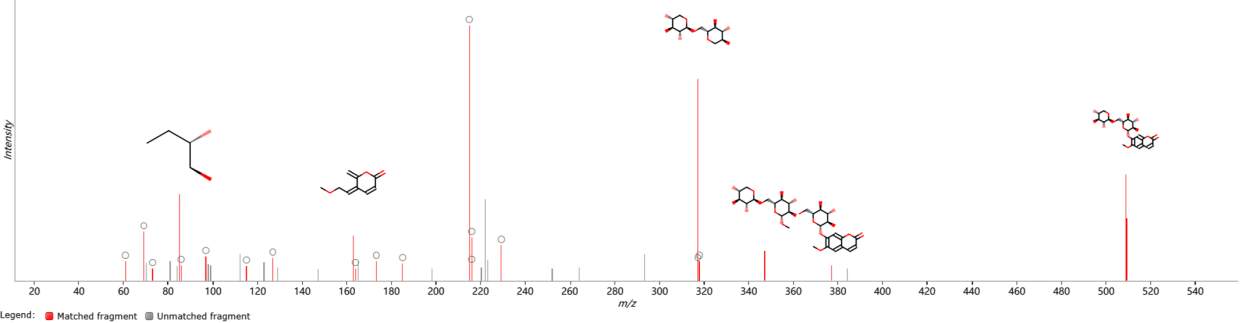 |
| **fabiatrin 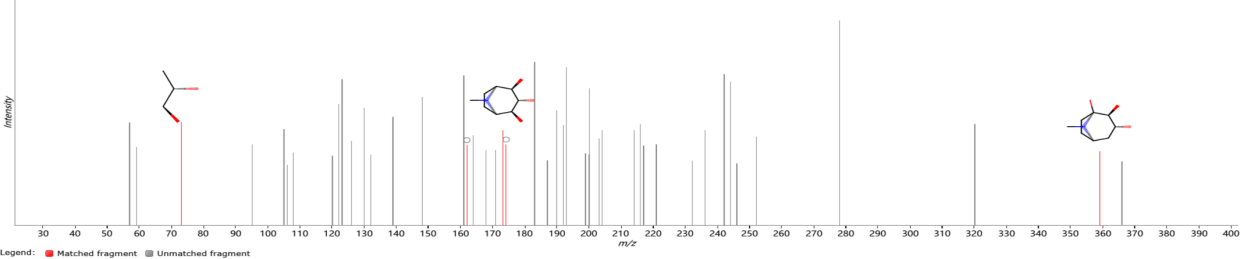 N-Methylcalystegine B2 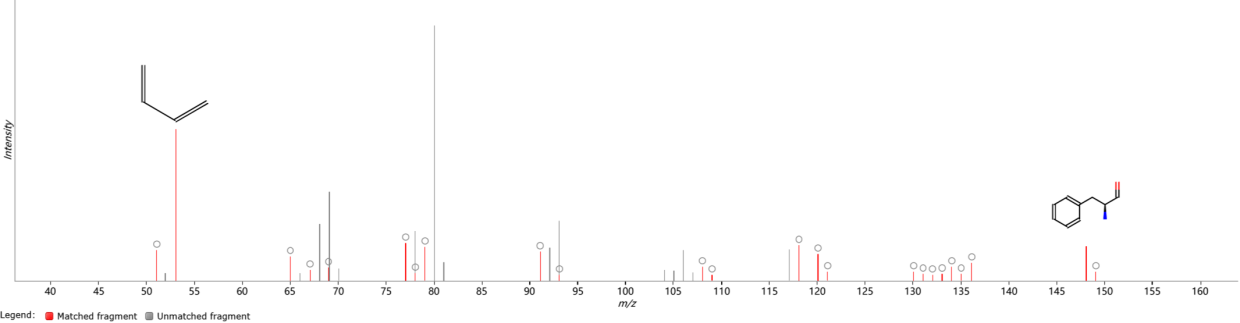 Phenylalanine 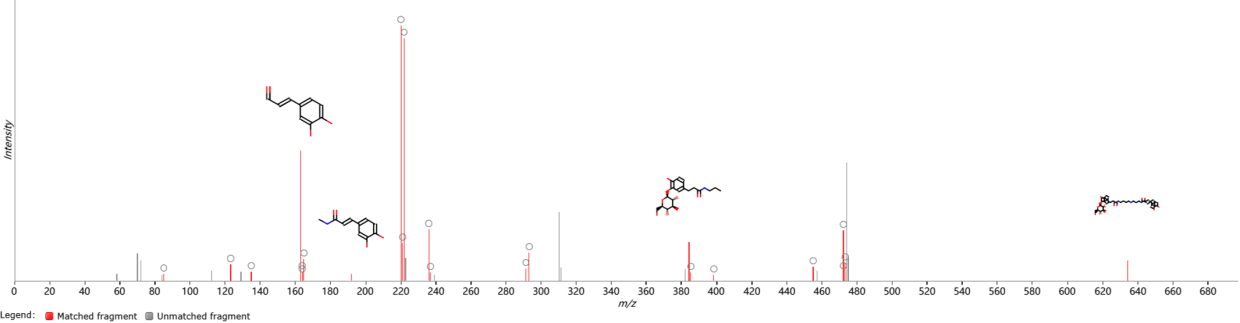 lycibarbarspermidine C 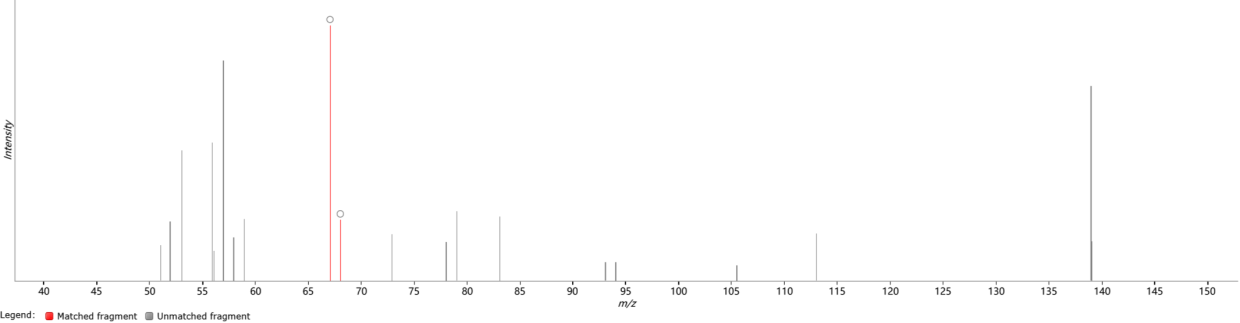 Ethyl 2-pyrrolecarboxylate 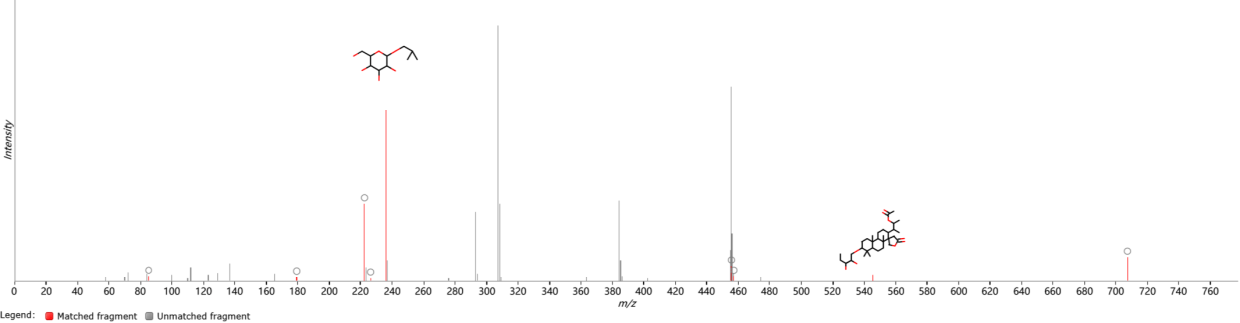 Hovenidulcioside A2 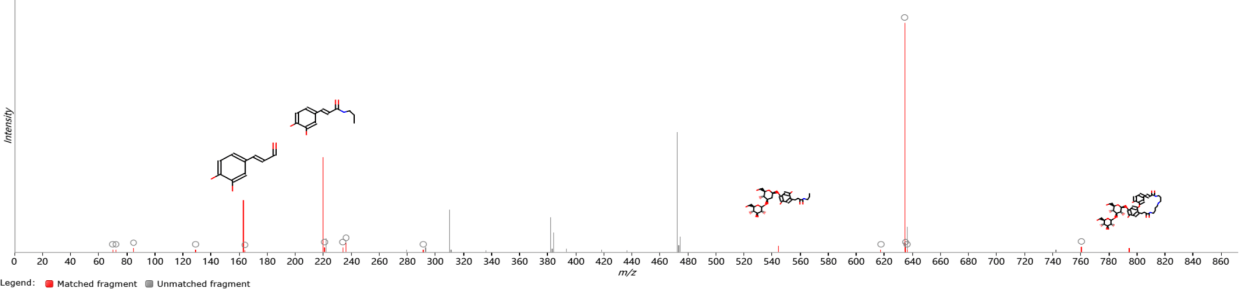 Lycibarbarspermidine O** |


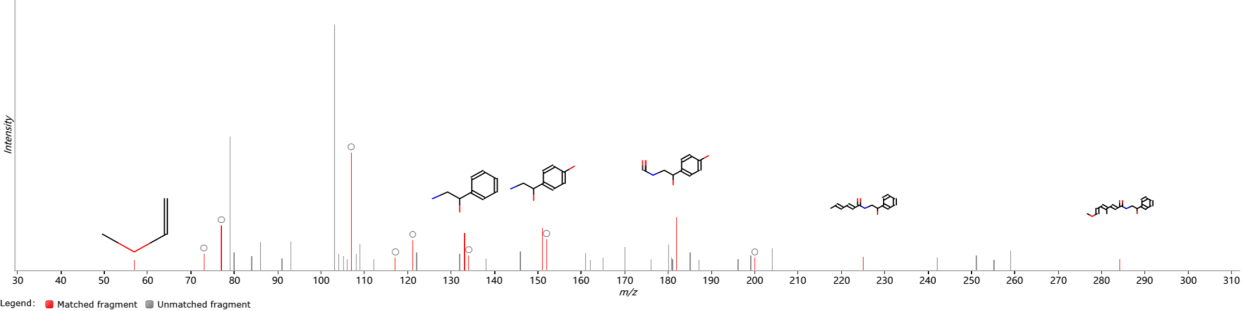
 **N-trans-Feruloyloctopamine**
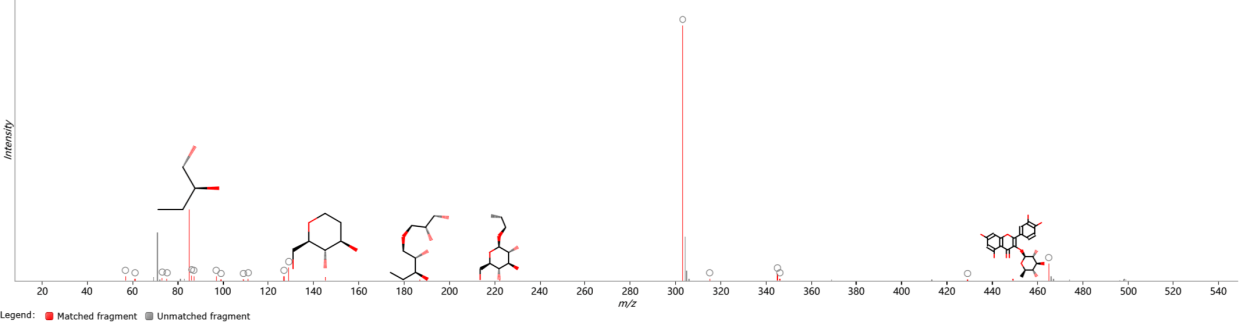
 **Quercetin 3-O-glucosyl-rutinoside**


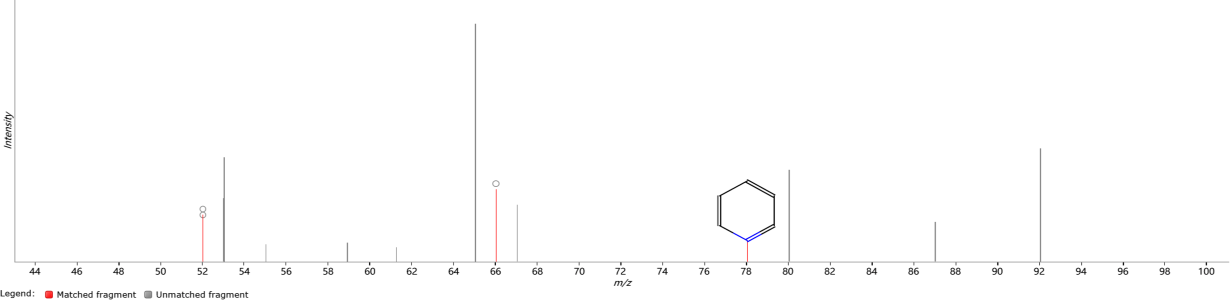
**5-Hydroxy-2-pyridyl-methyl ketone**


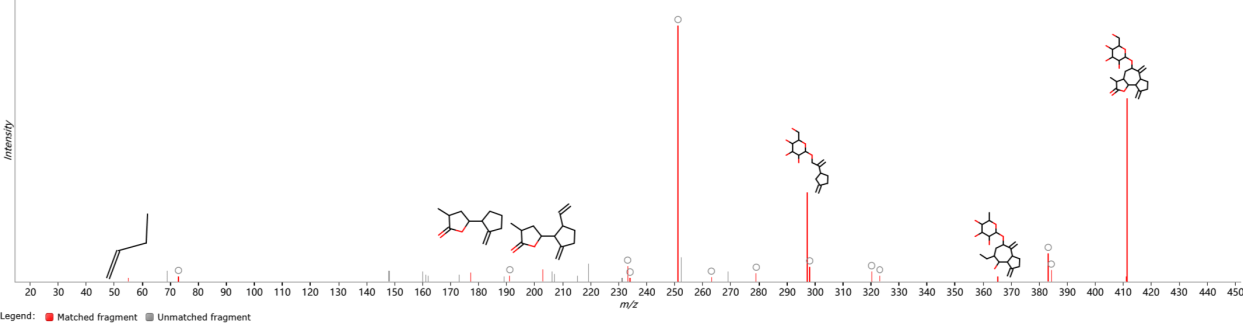
 **Scorzoside**
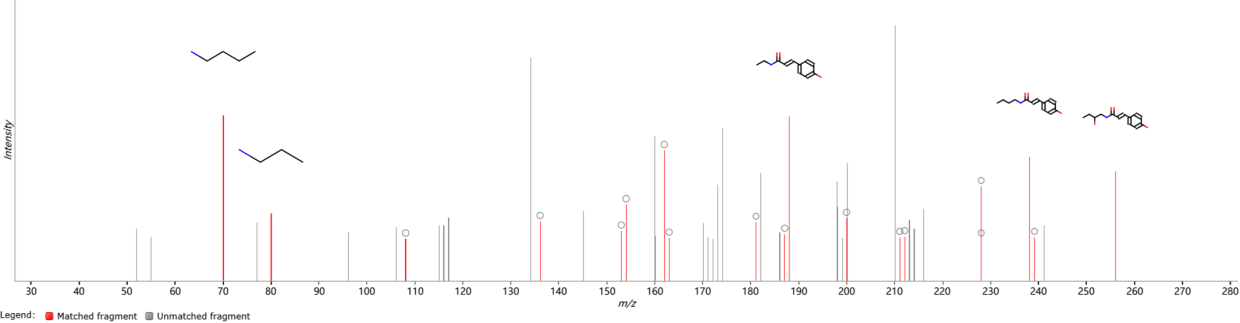
 **4-Coumaroyl-2-hydroxyputrescine**
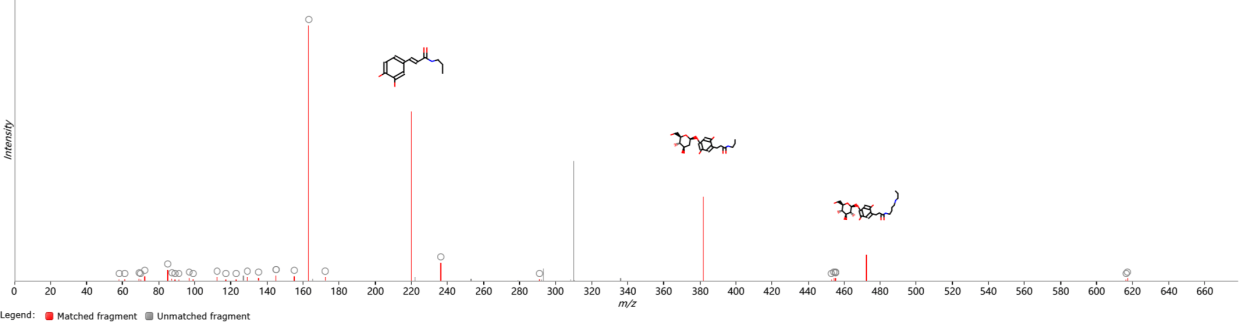
 **Lycibarbarspermidine N**
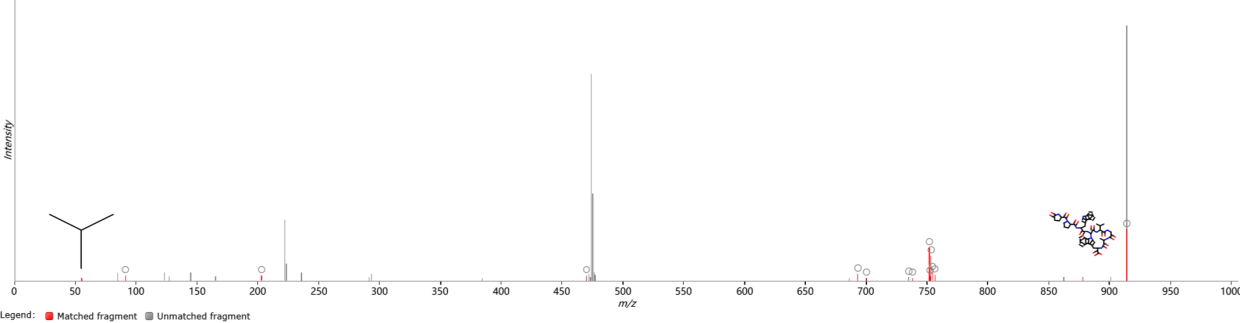
 **Lyciumins B**
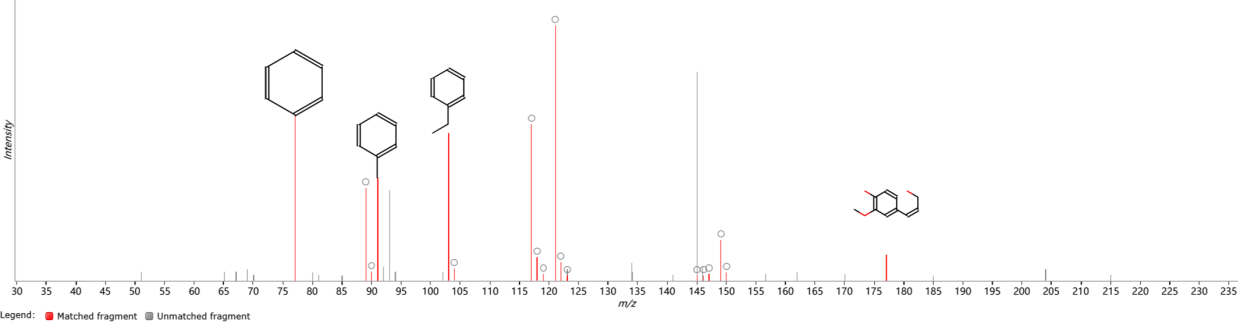
 **N-cis-feruloyl-tyramine**
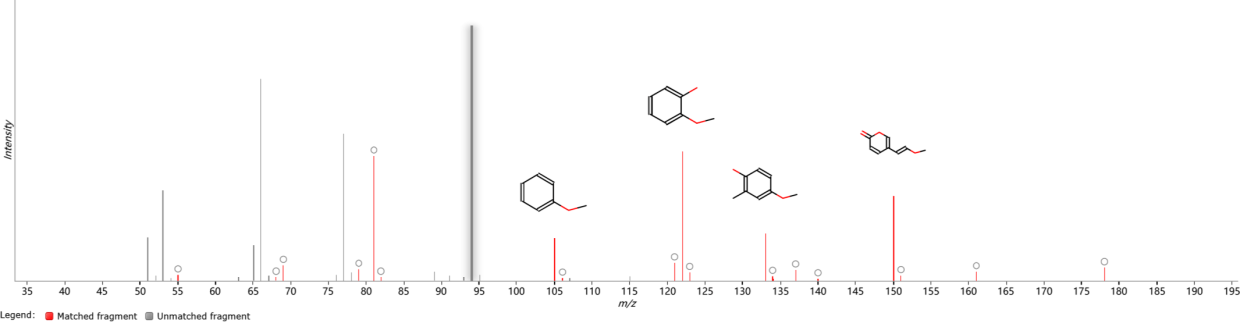
 **Scopoletin**


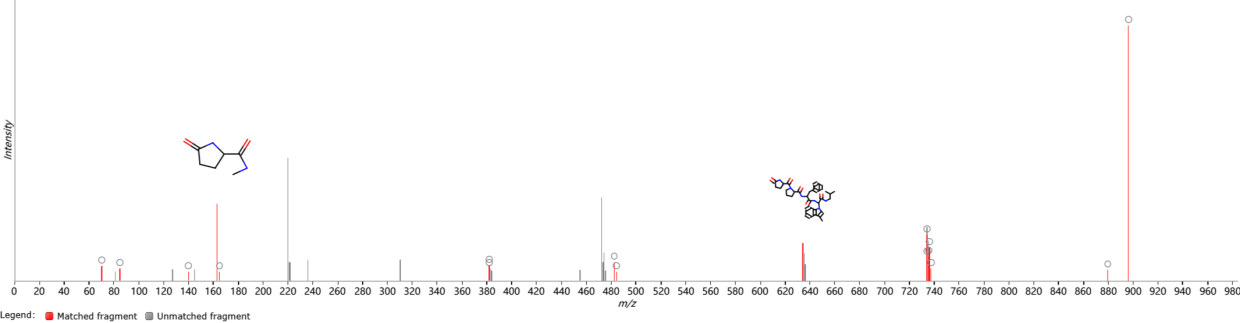
 **Lyciumins A**
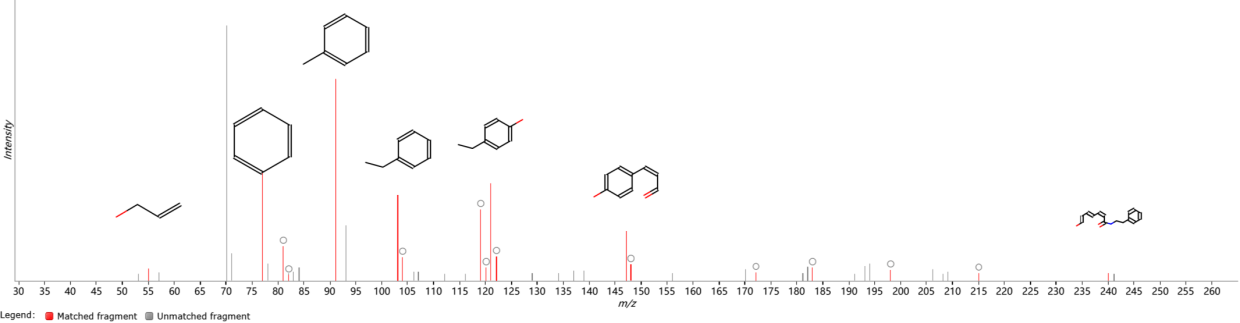
 **N-Z-p-coumaroyl-tyramine**
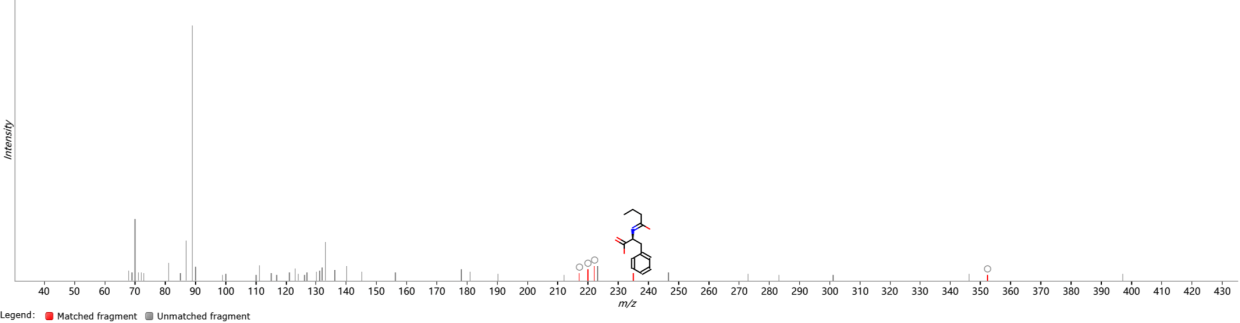
 **N-Docosahexaenoyl phenylalanine**
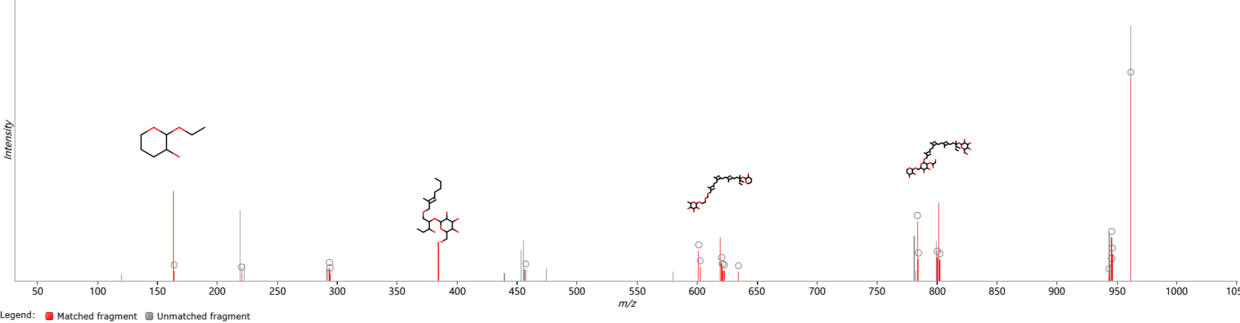
 **Lyciumoside VII**
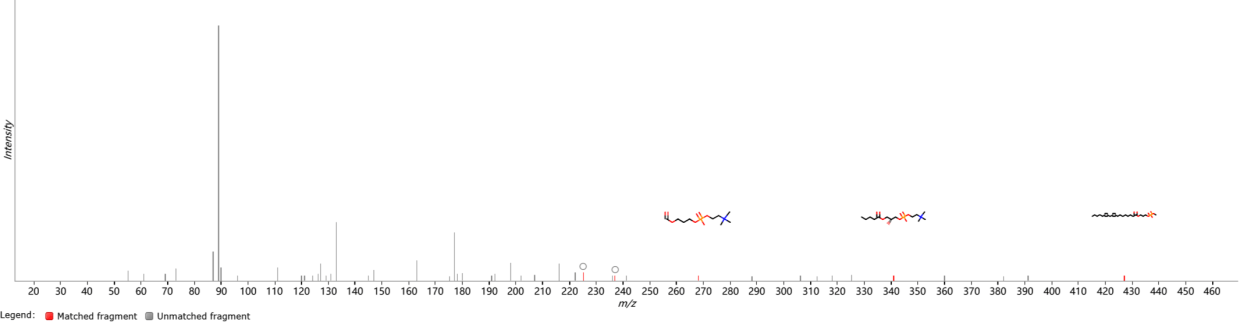
 **LysoPC(18:2(9Z,12Z)/0:0)**
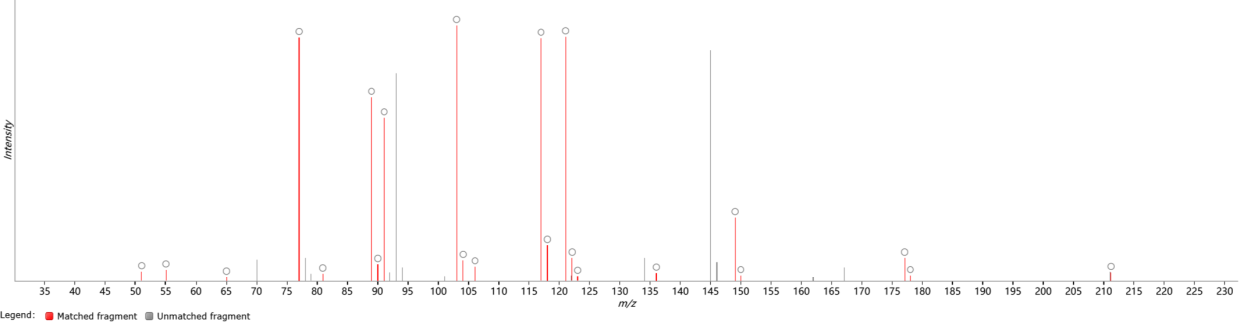
 **N-trans-feruloyl-tyramine**
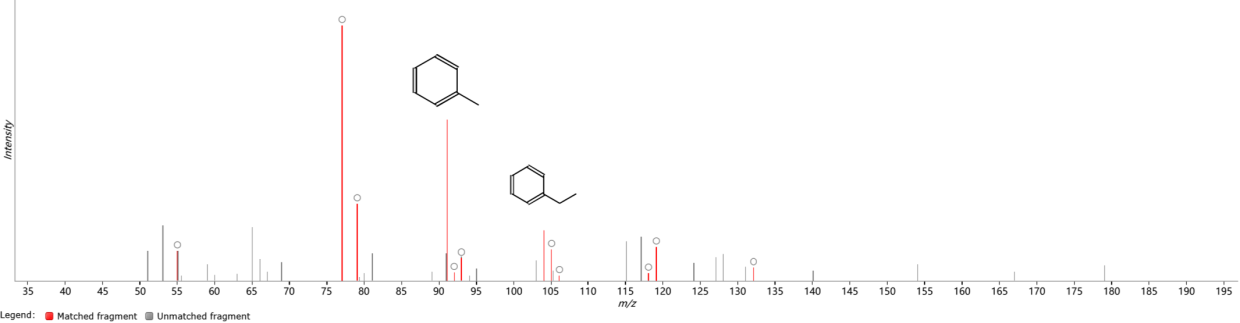
 **5-Phenylvaleric acid**
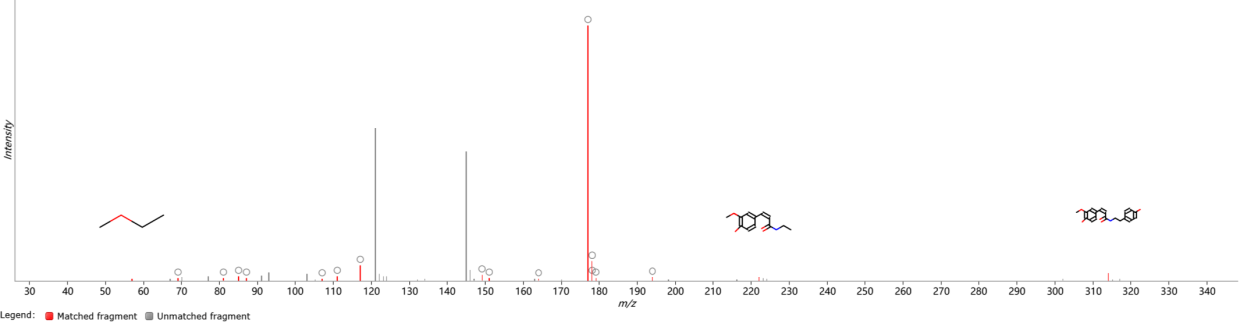
 **N-Z-feruloyl-4-O-(β-D-glucopyranosyl) tyramine**
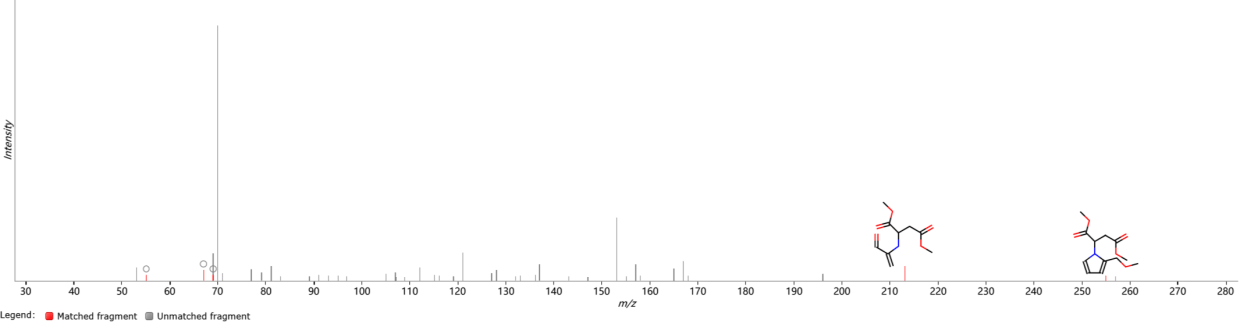
 **Dimethyl 2-[2-formyl-5-(methoxymethyl)-1H-pyrrol-1-yl]-butanedioate**
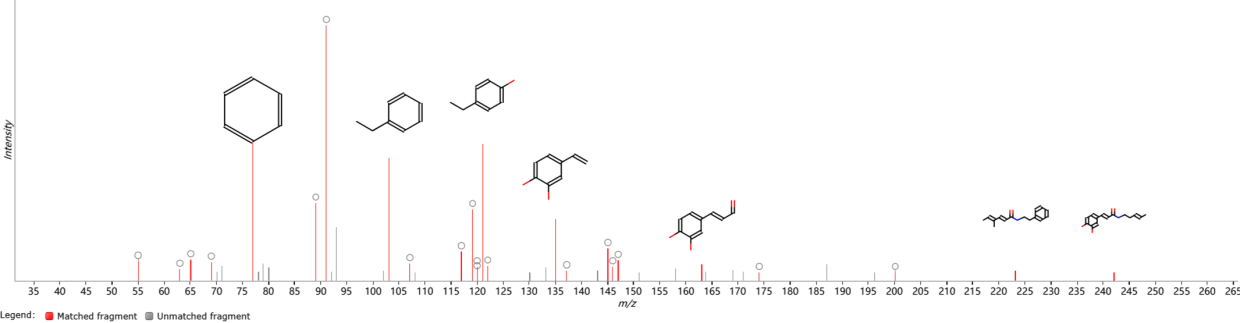
 **N-cis-Caffeoyltyramine**
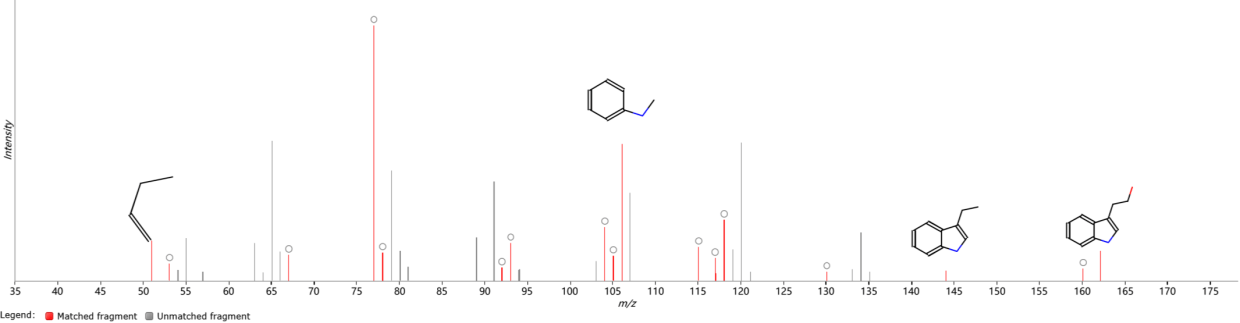
 **Tryptophol**
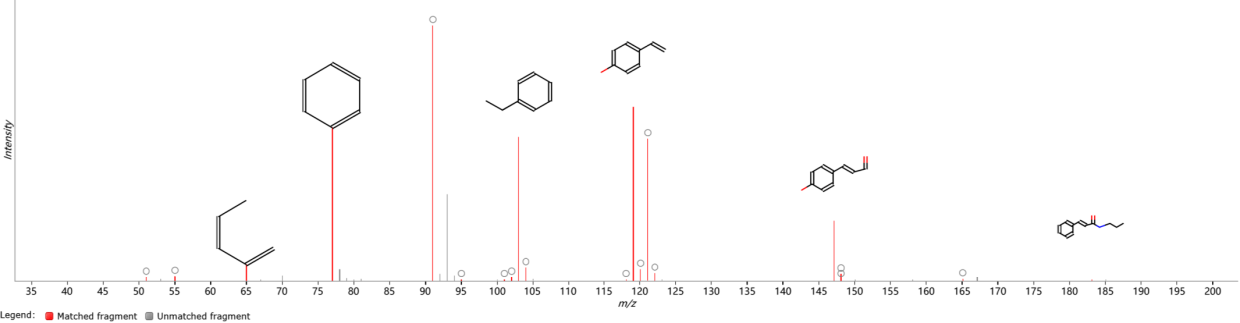
 **N-E-p-coumaroyl-tyramine**
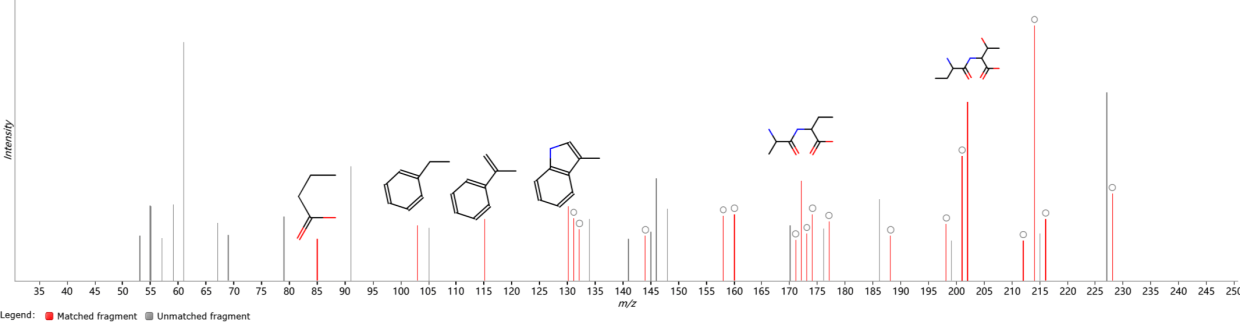
 **Threony-tryptophan**
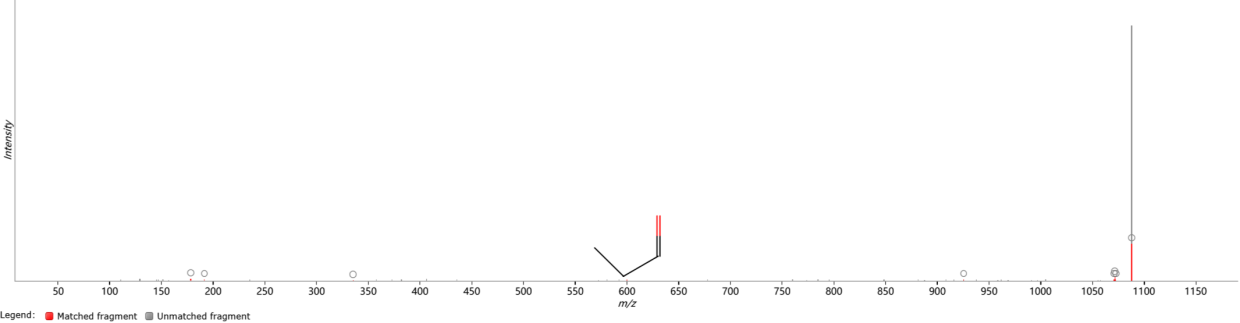
 **Lyrium spermidine A**
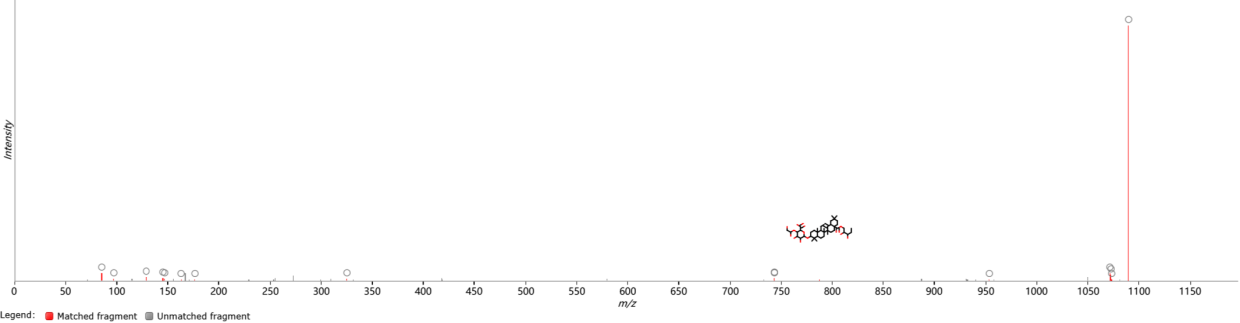
 **Araloside C**
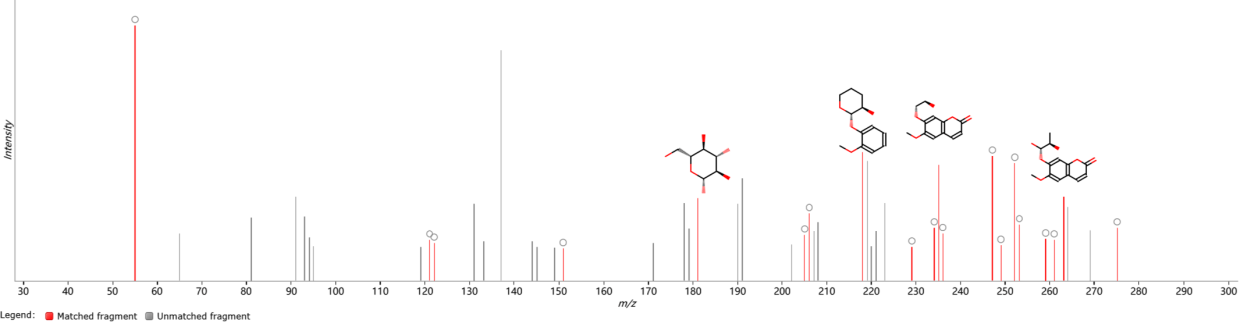
 **Scopolin**
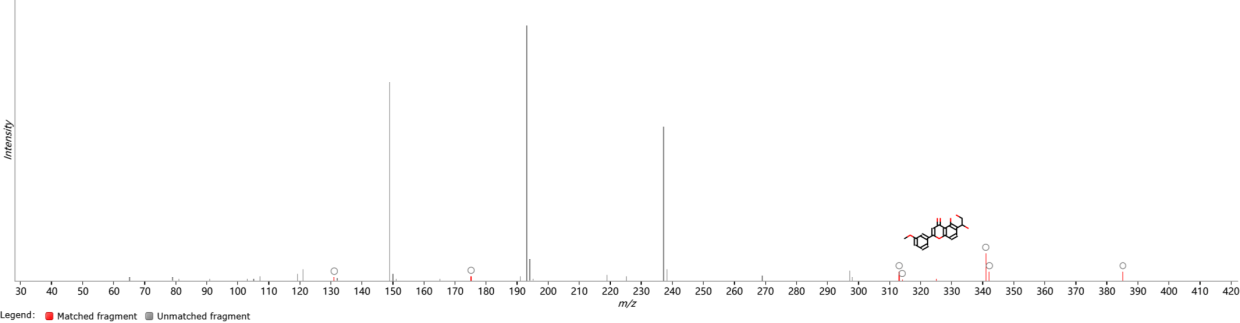
 **6-(3,4-dihydroxy-6methy-5-oxooxan-2-yl)-5,7-dihydroxy-2-(3-methoxyphenyl)-4H-chromen-4-one**
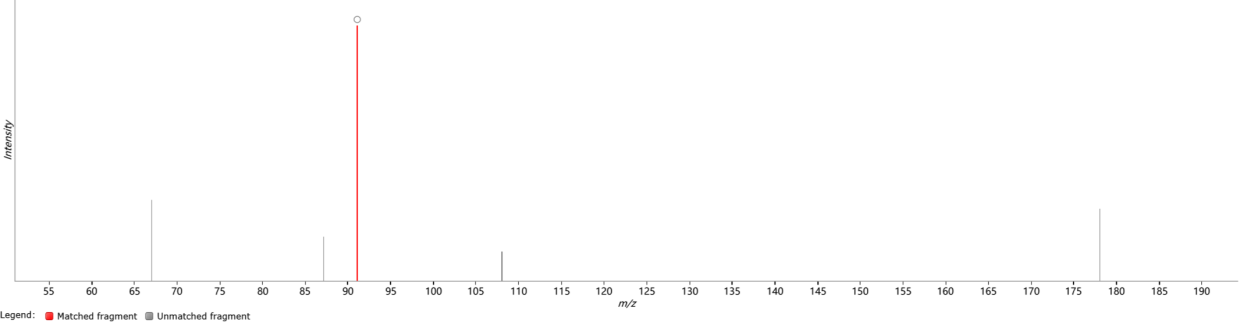
 **N, N-Diethylbenzeneacetamide**
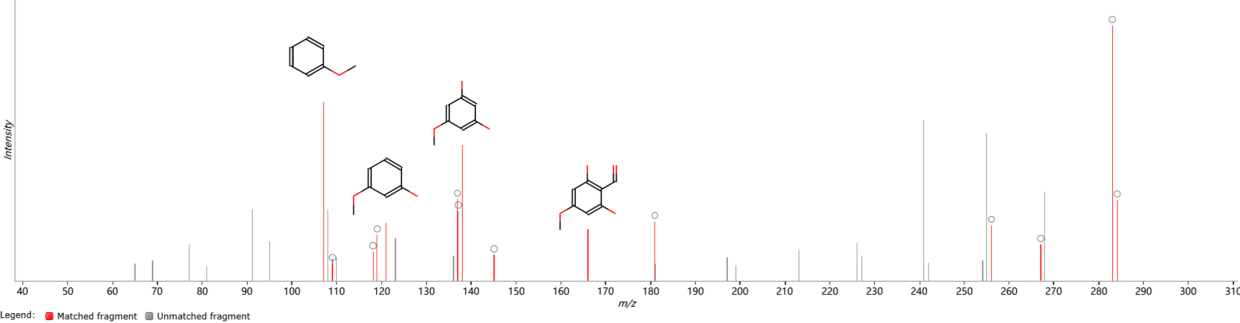
 **Apigenin 7,4'-dimethyl ether**
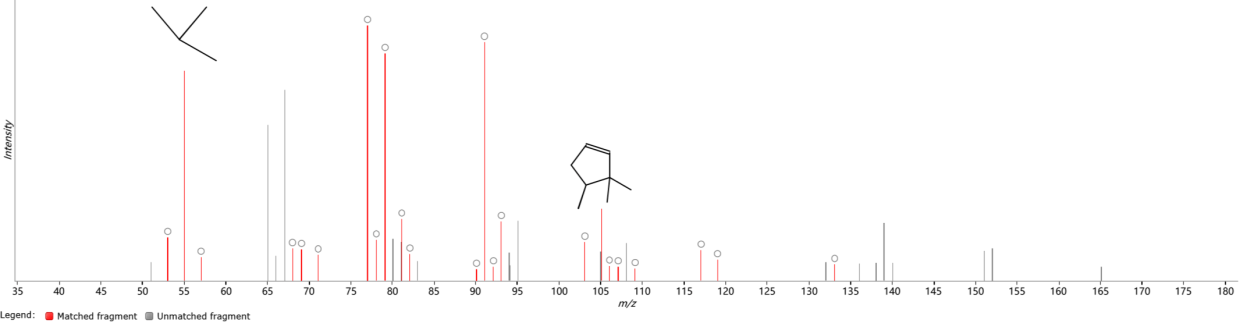
 **2,4,5,7alpha-Tetrahydro-1,4,4,7a-tetramethyl-1H-inden-2-ol**


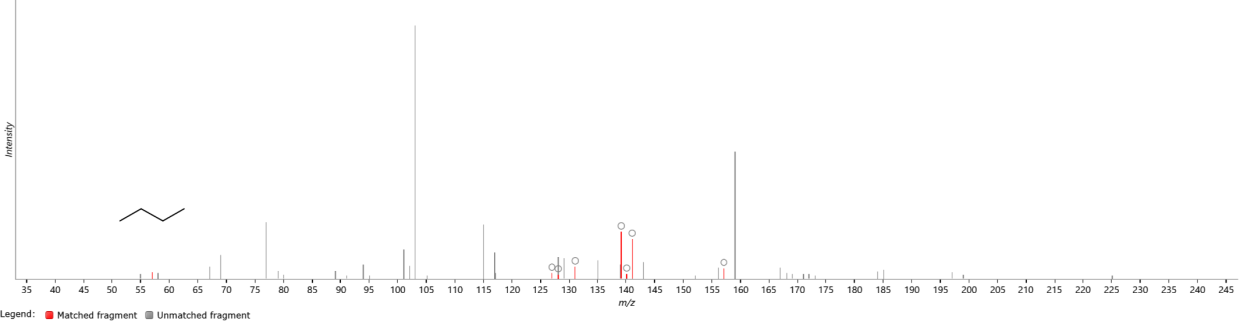
 **Tridecanoylglycine**


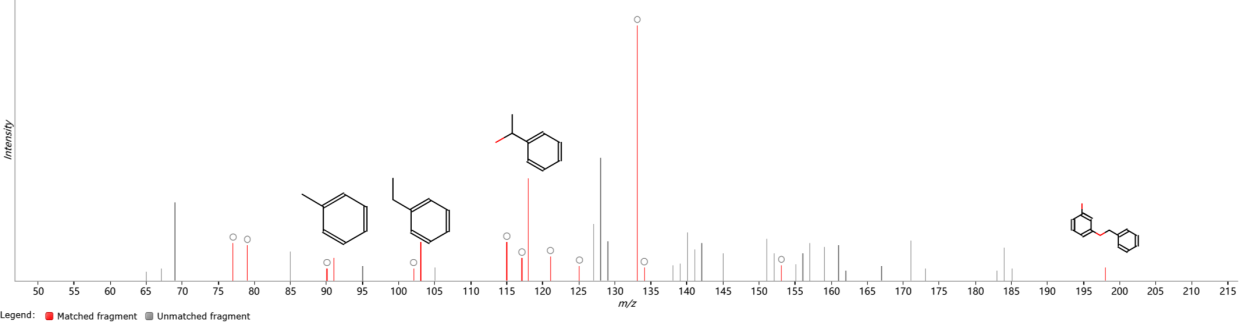
 **2-phenyl-3,4-dihydro-2H-1-benzopyran-3,5,7-triol**


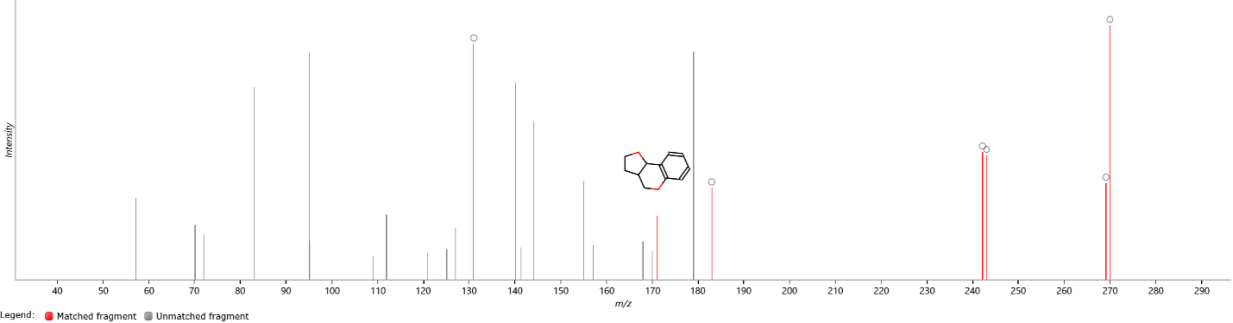


**Maackiain**


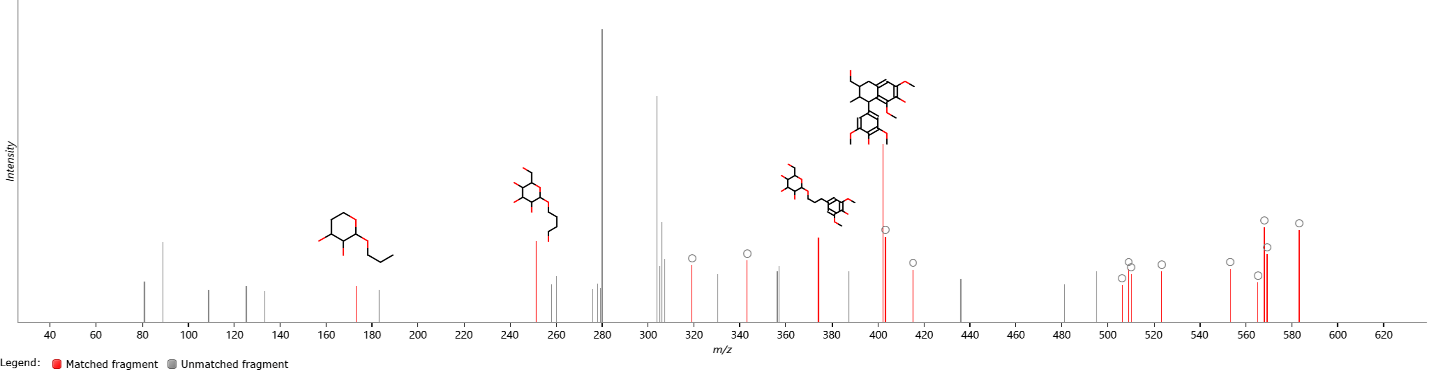


**(+)-Lyoniresinol-3α-O-β-D-Glucopyranoside**
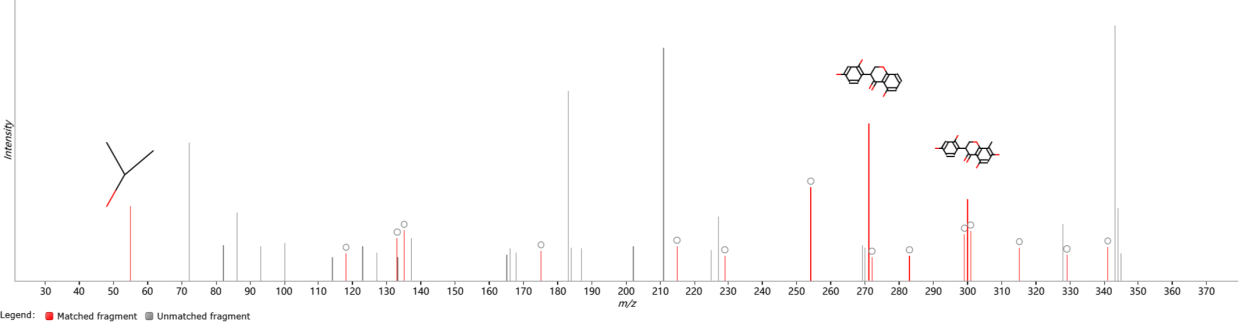


**Cyclokievitone hydrate**


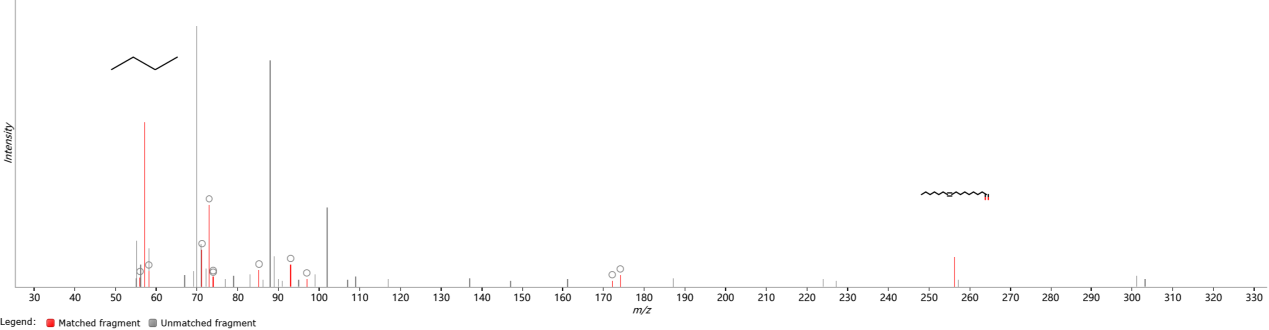
**N-(14-Methylhexadecanoyl)pyrrolidine**

**
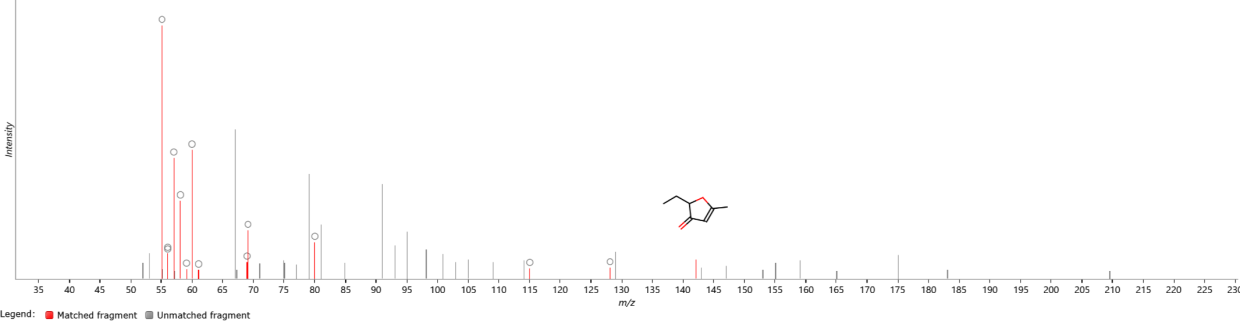
**

**Cepanone**


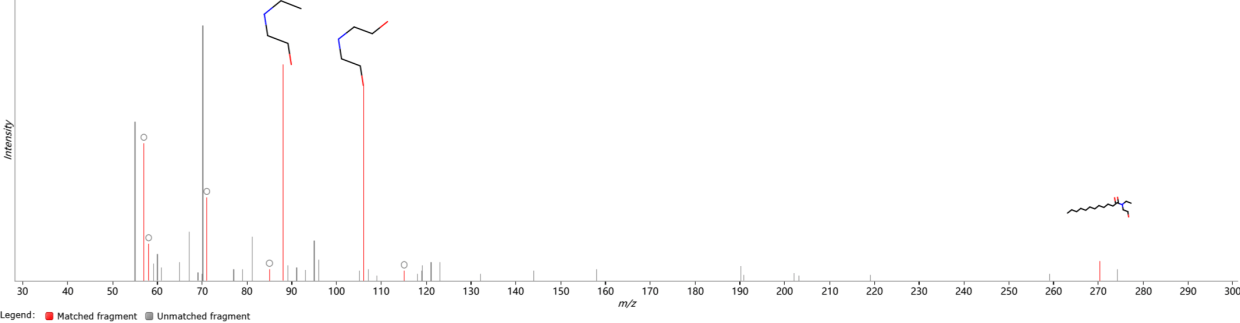
 **N,N-bis(2-hydroxyethyl)dodecanamide**
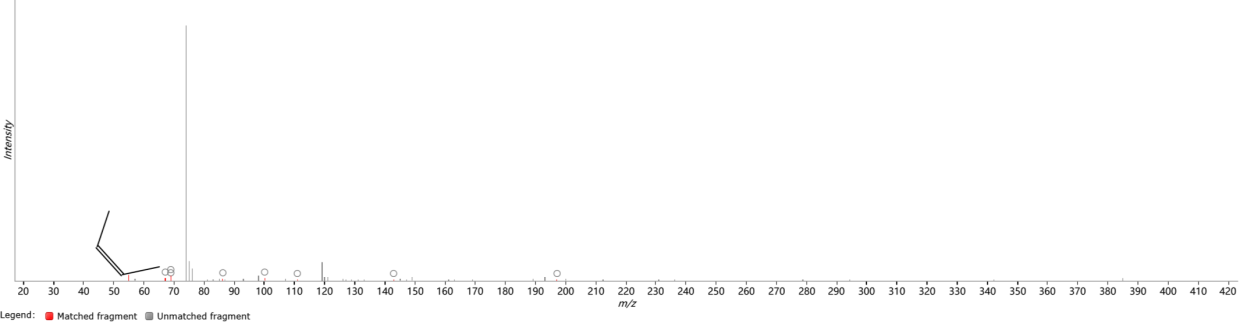
 **Withanolide A**
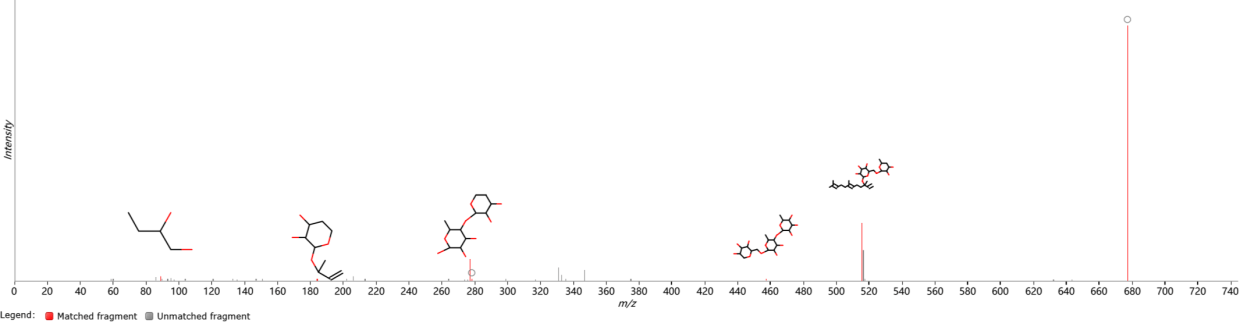
 **(S)-Nerolidol 3-O-[a-L-rhamnopyranosyl-(1->4)-a-L-rhamnopyranosyl-(1->6)-b-D-glucopyranoside]**
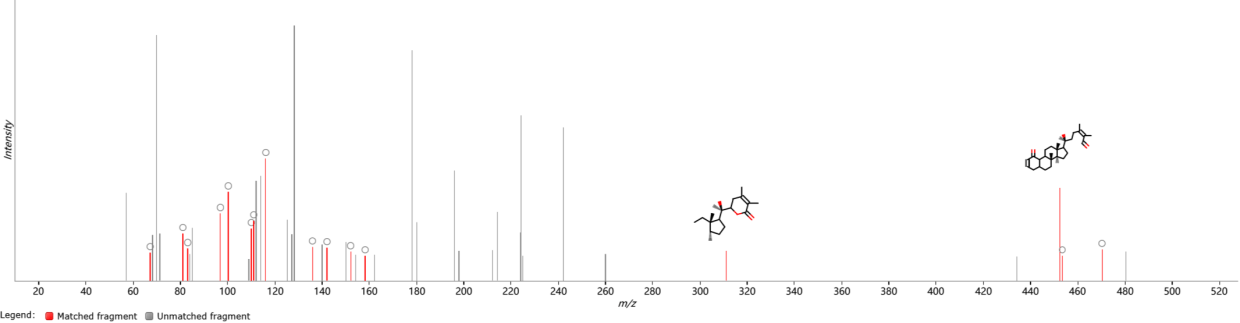
 **Lyciumsubstanz A**
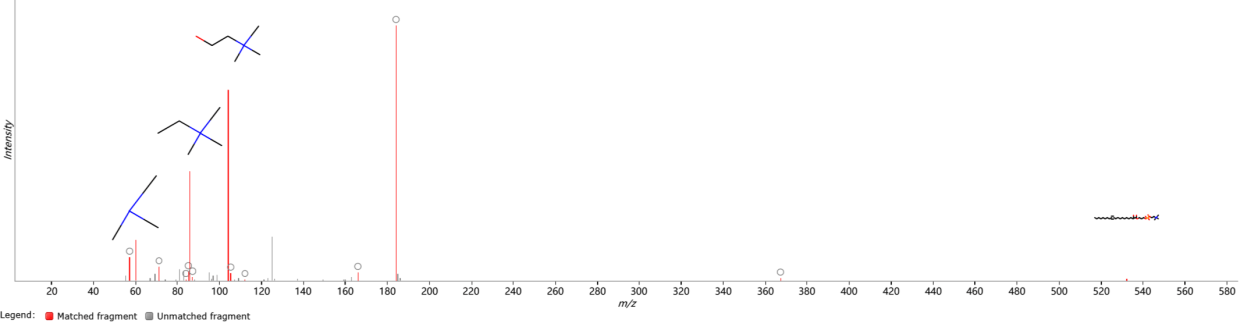
 **LysoPC(20:1(11Z)/0:0)**
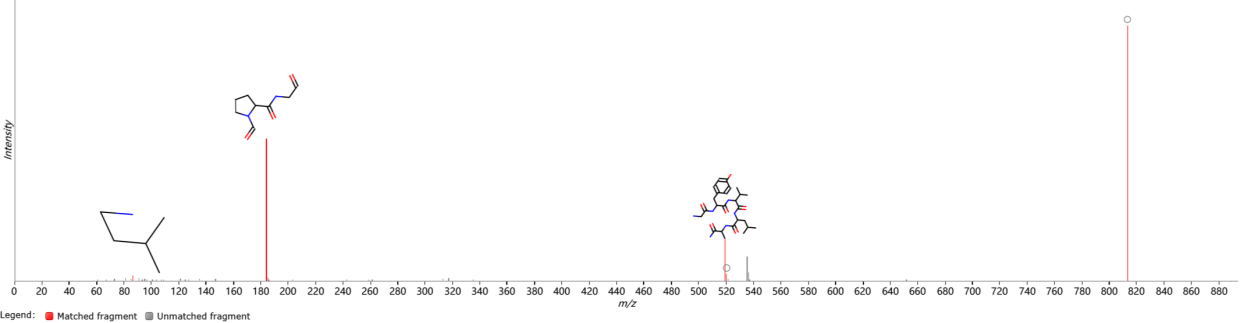
 **Glabrin C**
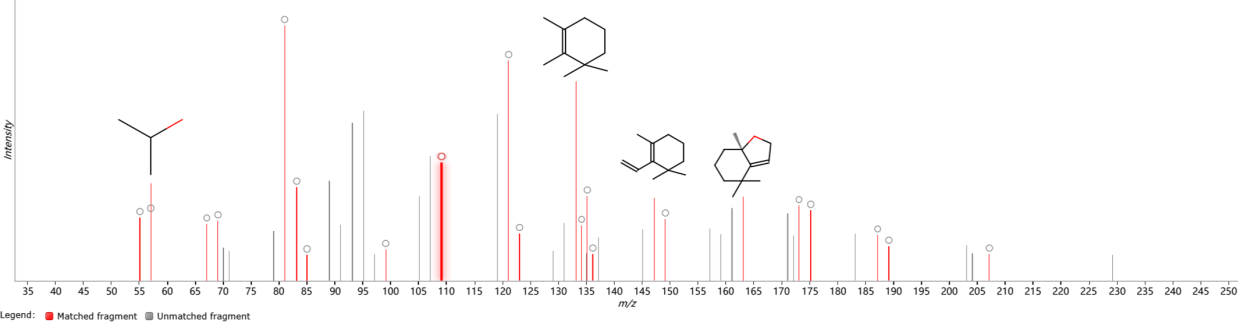
 **Mutatoxanthin**
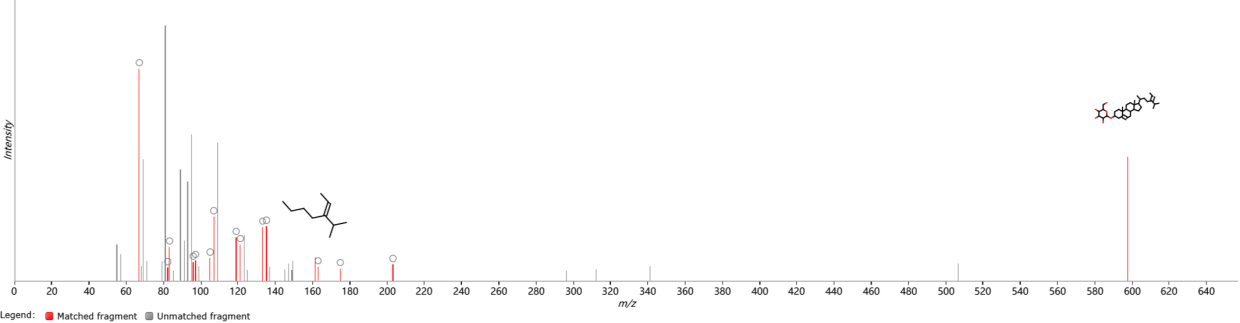
 **Isofucosterol glucoside**


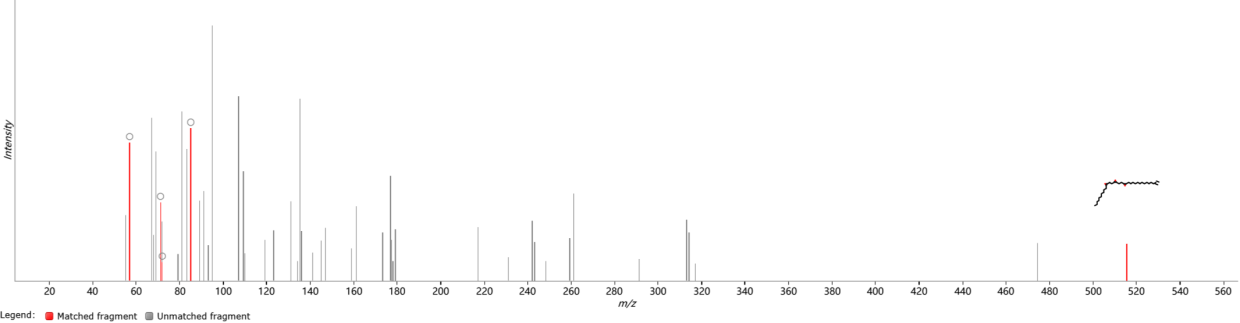
 **Tripoxyrollin**
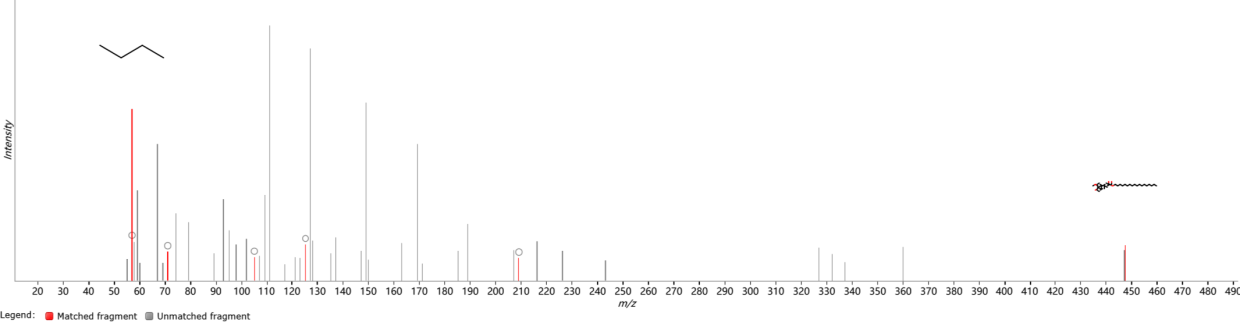
 **Stearyl ferulate**
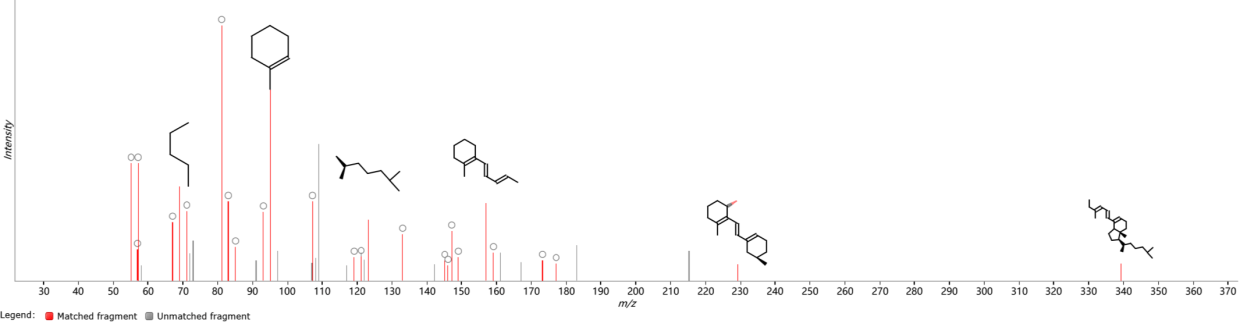
 **25-Hydroxytachysterol3**
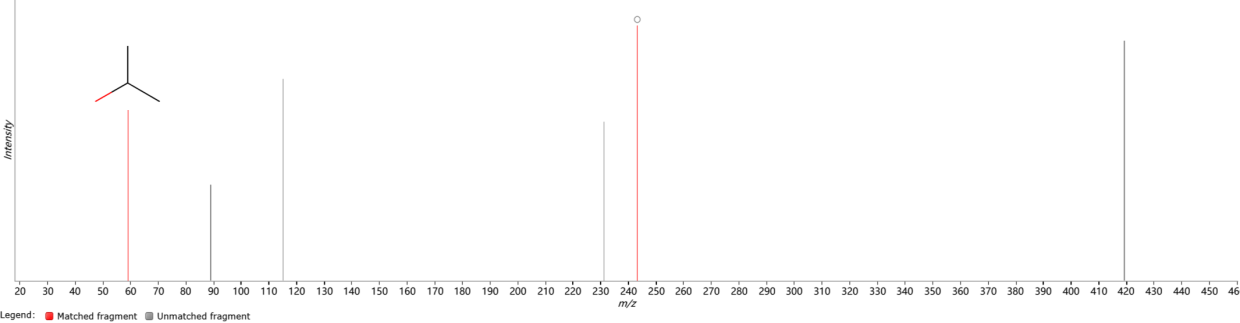
 **Beta-Cryptoxanthin**
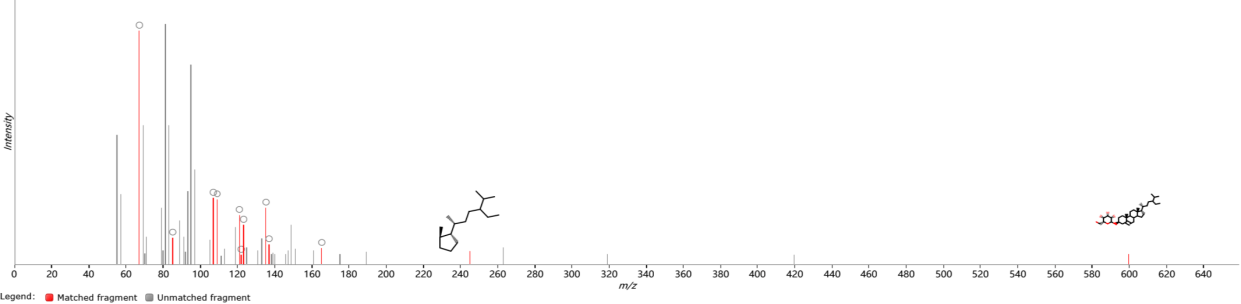
**β-Sitosterol β-D-glucopyranoside**


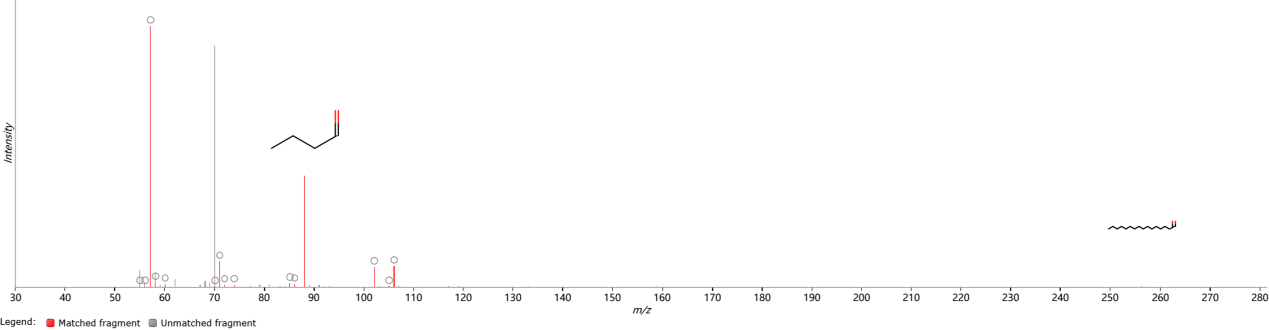
**Palmitic acid**


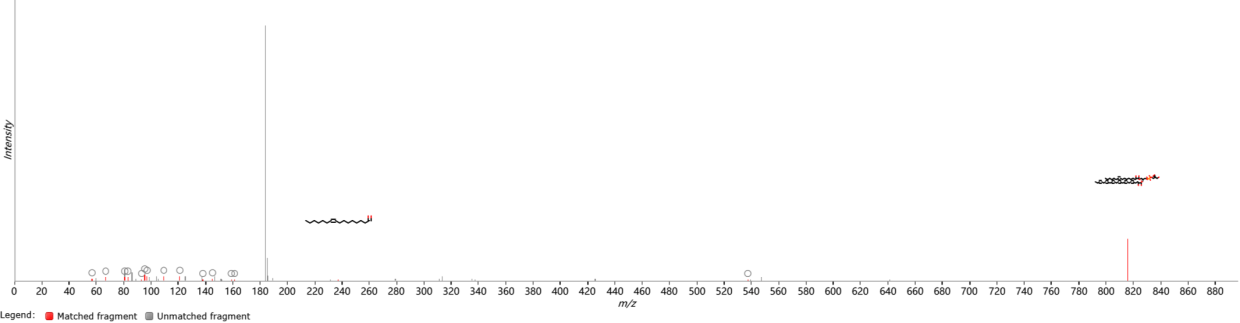
 **Goyaglycoside h**
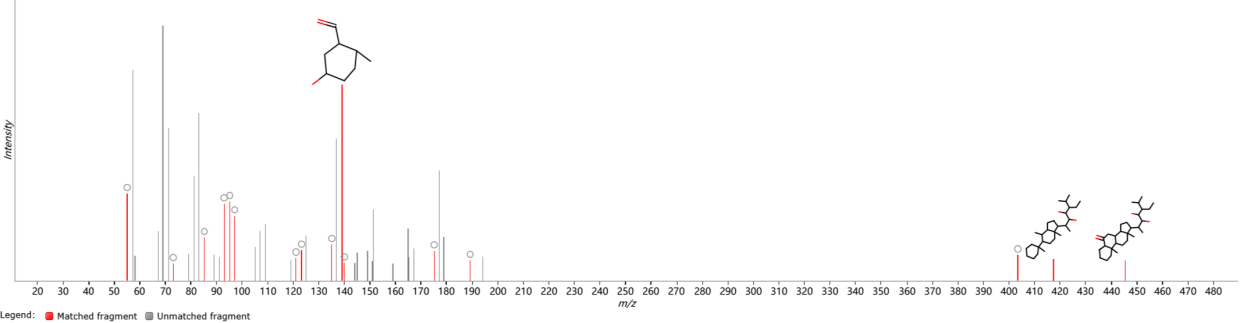
 **(3beta,22R,23R,24S)-3,22,23-Trihydroxystigmastan-6-one**
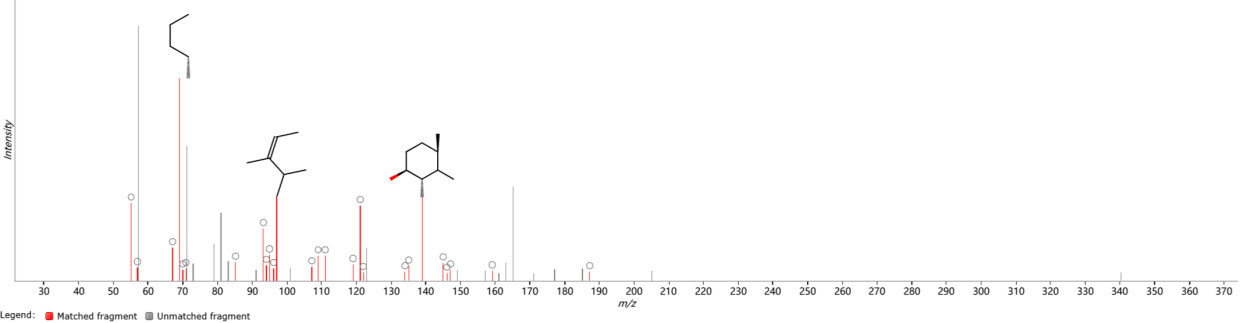
 **Citrostadienol**
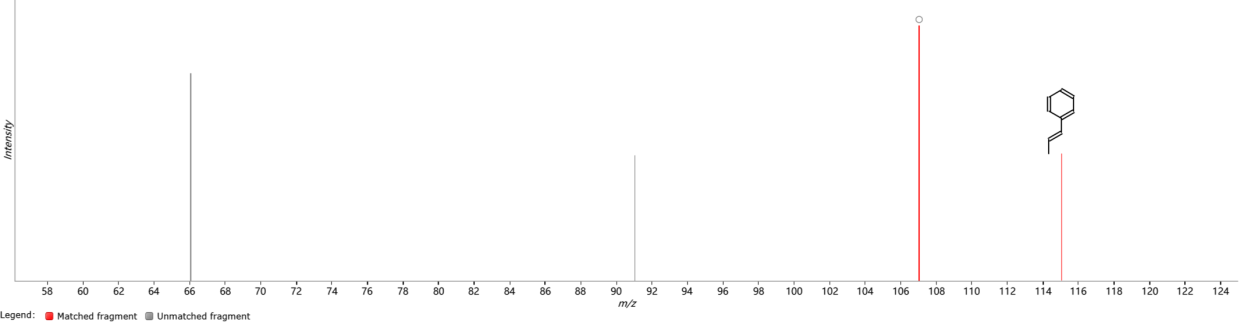
 **3-(4-Methoxyphenyl)-2-propen-1-ol**
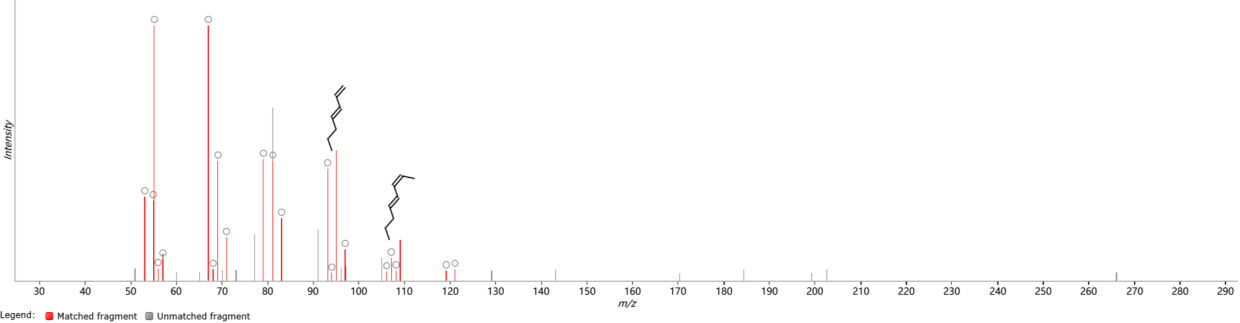
 **(9S,10E,12Z,15Z)-9-Hydroxy-10,12,15-octadecatrienoic acid**
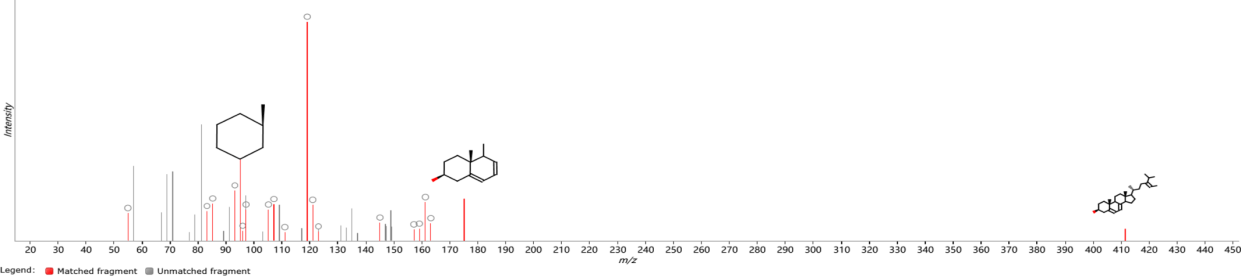
 **5-Dehydroavenasterol**

**Fig. S8**


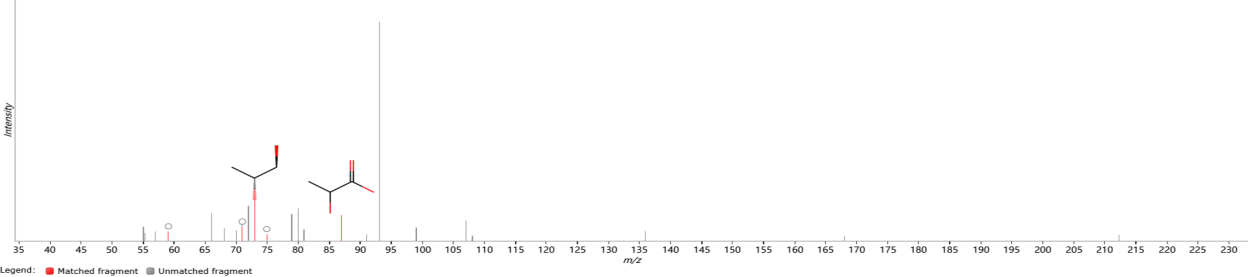
**2,3,4,5,6,7-Hexahydroxyheptanoic acid**

**L-Glutamine**  **Isocitric acid**  **mannose**  **Dehydroascorbic acid**  **Oxalic acid**  **p-Hydroxybenzoic acid**  **E)-2',4,4'-Trihydroxy-3-prenylchalcone**  **b-D-Glucuronopyranosyl-(1->3)-a-D-galacturonopyranosyl-(1->2)-L-rhamnose** **Caffeic acid**  **N1-Caffeoyl-N3-dihydrocaffeoyl spermidine**  **2-Formyl-5-hydroxymethylpyrrole** **lycibarbarspermidine B**  **lycibarbarspermidine F**  **Lycibarbarphenylpropanoids C**  **Methyl cholorogenate**  **10-Hydroxy-2,8-decadiene-4,6-diynoic acid**  **Indole-3-acetylglutamic acid**  **Melongoside P**  **Phloretic acid**

**5-Hydroxy-3,3',7,8-tetramethoxy-4',5'-methylenedioxyflavone** **Cannabisin F**

**LysoPC(18:2(9Z,12Z))**

**3-Hydroxy-6,8-dimethoxy-7(11)-eremophilen-12,8-olide**  **Lyciumsubstanz B**  **18-Dehydroursolic acid 3-arabinoside**  **Asparagoside A**

**Oleic acid** **Sorbitan stearate**  **Isofucosterol glucoside** **7',8'-Dihydro-8'-hydroxycitraniaxanthin** **Stearic acid** **PGP(16:0/18:0)**  **Solavetivone**

**PS(15:0/18:1(11Z))**  **Momordicoside B**

**Soyasapapogenol B 24-O-b-D-glucoside** **4,6-Pentacosanedione**  **Arachidic acid**  **Stigmasterol**

**1,2-Bis(9Z,12Z)-octadecadienoyl-sn-glycero-3-phospho-1D-myo-inositol** **Cerebronic acid**  **Oleanolic acid 3-[rhamnosyl-(1->4)-glucosyl-(1->6)-glucoside]**

**Cycloartanol**

**behenic acid**

**24-Ethyl-4α-methylcholesta7,24-dien-3β-ol**  **Solanocardinol**

**Fig. S9**

B

C

E

F

G

A

D

**Fig. S10**

A

B

E

D

C

F

G

**Fig. S11**

**Fig. S12**
