# Supplementary material for: A comparative UHPLC-QTOF-MS/MS-based metabolomics approach reveals the metabolite profiling of wolfberry sourced from different geographical origins
Source: Food Chem X. 2024 Feb 10;21:101221. doi: 10.1016/j.fochx.2024.101221 (PMC10877177; doi:10.1016/j.fochx.2024.101221)
Supplement: Supplementary data 5 [file mmc5.pdf]

**Tab.S5 KEGG pathway analysis of NX vs NNX**

| pathway | enrichment | p value | enrichment Ratio |
|---|---|---|---|
| Tryptophan metabolism | 1.57024772 | 0.0269 | 37.17472119 |
| Aminoacyl-tRNA biosynthesis | 1.501689446 | 0.0315 | 31.74603175 |

**Tab.S5 KEGG pathway analysis of NX vs GS**

| pathway | enrichment | p value | enrichment Ratio |
|---|---|---|---|
| Citrate cycle (TCA cycle) | 3.021363052 | 0.000952 | 38.38771593 |
| Alanine, aspartate and glutamate metabolisr | 2.725842151 | 0.00188 | 27.43484225 |
| Glyoxylate and dicarboxylate metabolism | 2.609064893 | 0.00246 | 24.00960384 |
| Aminoacyl-tRNA biosynthesis | 2.258848401 | 0.00551 | 16 |
| D-Glutamine and D-glutamate metabolism | 1.809668302 | 0.0155 | 64.1025641 |
| Nitrogen metabolism | 1.809668302 | 0.0155 | 64.1025641 |
| Arginine biosynthesis | 1.443697499 | 0.036 | 27.39726027 |
| Butanoate metabolism | 1.41453927 | 0.0385 | 25.57544757 |
| Propanoate metabolism | 1.232102384 | 0.0586 | 16.69449082 |
| Pyrimidine metabolism | 1.009217308 | 0.0979 | 9.803921569 |
| Tryptophan metabolism | 0.987162775 | 0.103 | 9.345794393 |
| Purine metabolism | 0.798602876 | 0.159 | 5.917159763 |

| Tab.S5 KEGG pathway analysis of NX vs QH | | | |
|---|---|---|---|
| **pathway** | **enrichment** | **p value** | **enrichment Ratio** |
| Aminoacyl-tRNA biosynthesis | 3.019088062 | 0.000957 | 32 |
| Phenylalanine, tyrosine and tryptophan biosynthes | 2.283996656 | 0.0052 | 191.9385797 |
| Phenylalanine metabolism | 1.886056648 | 0.013 | 76.92307692 |
| Tryptophan metabolism | 1.278189385 | 0.0527 | 18.72659176 |

**Tab.S5 KEGG pathway analysis of NX vs XJ**

| pathway | enrichment | p value | enrichment Ratio |
|---|---|---|---|
| Biosynthesis of unsaturated fatty acids | 3.411168274 | 0.000388 | 18.29268293 |
| Citrate cycle (TCA cycle) | 2.488116639 | 0.00325 | 21.95389682 |
| Linoleic acid metabolism | 1.645891561 | 0.0226 | 43.85964912 |
| Butanoate metabolism | 1.177178355 | 0.0665 | 14.61988304 |
| Fructose and mannose metabolism | 1.056505484 | 0.0878 | 10.97694841 |
| Propanoate metabolism | 1 | 0.1 | 9.523809524 |
| Galactose metabolism | 0.931814138 | 0.117 | 8.130081301 |
| Alanine, aspartate and glutamate metabolism | 0.91721463 | 0.121 | 7.8125 |
| Glyoxylate and dicarboxylate metabolism | 0.863279433 | 0.137 | 6.849315068 |
| Amino sugar and nucleotide sugar metabolism | 0.804100348 | 0.157 | 5.917159763 |
| Tryptophan metabolism | 0.761953897 | 0.173 | 5.347593583 |
| Aminoacyl-tRNA biosynthesis | 0.698970004 | 0.2 | 4.566210046 |

| Tab.S5 KEGG pathway analysis of XJ vs GS | | | |
|---|---|---|---|
| **pathway** | **enrichment** | **p value** | **enrichment Ratio** |
| Biosynthesis of unsaturated fatty acids | 5.071604148 | 0.00000848 | 24.3902439 |
| Glyoxylate and dicarboxylate metabolism | 2.081969663 | 0.00828 | 13.69863014 |
| Linoleic acid metabolism | 1.645891561 | 0.0226 | 43.85964912 |
| D-Glutamine and D-glutamate metabolism | 1.567030709 | 0.0271 | 36.63003663 |
| Nitrogen metabolism | 1.567030709 | 0.0271 | 36.63003663 |
| Arginine biosynthesis | 1.206209615 | 0.0622 | 15.67398119 |
| Citrate cycle (TCA cycle) | 1.056505484 | 0.0878 | 10.97694841 |
| Fructose and mannose metabolism | 1.056505484 | 0.0878 | 10.97694841 |
| Galactose metabolism | 0.931814138 | 0.117 | 8.130081301 |
| Alanine, aspartate and glutamate metabolism | 0.91721463 | 0.121 | 7.8125 |
| Amino sugar and nucleotide sugar metabolism | 0.804100348 | 0.157 | 5.917159763 |
| Fatty acid elongation | 0.793174124 | 0.161 | 5.780346821 |
| Fatty acid degradation | 0.782516056 | 0.165 | 5.617977528 |
| Pyrimidine metabolism | 0.782516056 | 0.165 | 5.617977528 |
| Fatty acid biosynthesis | 0.707743929 | 0.196 | 4.672897196 |
| Aminoacyl-tRNA biosynthesis | 0.698970004 | 0.2 | 4.566210046 |
| Purine metabolism | 0.581698709 | 0.262 | 3.378378378 |

| Tab.S5 KEGG pathway analysis of XJ vs QH | | | |
|---|---|---|---|
| **pathway** | **enrichment** | **p value** | **enrichment Ratio** |
| Biosynthesis of unsaturated fatty acids | 6.202732459 | 6.27E-07 | 23.69668246 |
| Citrate cycle (TCA cycle) | 2.260427656 | 0.00549 | 17.09401709 |
| Linoleic acid metabolism | 1.537602002 | 0.029 | 34.12969283 |
| Butanoate metabolism | 1.07211659 | 0.0847 | 11.37656428 |
| Fructose and mannose metabolism | 0.950781977 | 0.112 | 8.547008547 |
| Propanoate metabolism | 0.896196279 | 0.127 | 7.407407407 |
| Galactose metabolism | 0.829738285 | 0.148 | 6.329113924 |
| Alanine, aspartate and glutamate metabolism | 0.815308569 | 0.153 | 6.097560976 |
| Glyoxylate and dicarboxylate metabolism | 0.761953897 | 0.173 | 5.319148936 |
| Amino sugar and nucleotide sugar metabolisr | 0.70333481 | 0.198 | 4.608294931 |
| Fatty acid elongation | 0.694648631 | 0.202 | 4.484304933 |
| Fatty acid degradation | 0.684029655 | 0.207 | 4.366812227 |
| Steroid biosynthesis | 0.655607726 | 0.221 | 4.06504065 |
| Fatty acid biosynthesis | 0.610833916 | 0.245 | 3.636363636 |

**Tab.S5 KEGG pathway analysis of GS vs QH**

| pathway | enrichment | p value | enrichment Ratio |
|---|---|---|---|
| D-Glutamine and D-glutamate metabolism | 2.107905397 | 0.0078 | 128.0409731 |
| Nitrogen metabolism | 2.107905397 | 0.0078 | 128.0409731 |
| Arginine biosynthesis | 1.739928612 | 0.0182 | 54.94505495 |
| Alanine, aspartate and glutamate metabolism | 1.442492798 | 0.0361 | 27.39726027 |
| Glyoxylate and dicarboxylate metabolism | 1.385102784 | 0.0412 | 23.98081535 |
| Pyrimidine metabolism | 1.299296283 | 0.0502 | 19.68503937 |
| Tyrosine metabolism | 1.26760624 | 0.054 | 18.28153565 |
| Aminoacyl-tRNA biosynthesis | 1.211124884 | 0.0615 | 16 |
| Purine metabolism | 1.081445469 | 0.0829 | 11.82033097 |
